# Knockout of the HMG domain of the porcine SRY gene causes sex reversal in gene-edited pigs

**DOI:** 10.1073/pnas.2008743118

**Published:** 2020-12-22

**Authors:** Stefanie Kurtz, Andrea Lucas-Hahn, Brigitte Schlegelberger, Gudrun Göhring, Heiner Niemann, Thomas C. Mettenleiter, Björn Petersen

**Affiliations:** ^a^Institute of Farm Animal Genetics, Friedrich-Loeffler-Institut, Mariensee, 31535 Neustadt am Rübenberge, Germany;; ^b^Institute of Human Genetics, Hannover Medical School, 30625 Hannover, Germany;; ^c^Clinic for Gastroenterology, Hepatology and Endocrinology, Hannover Medical School, 30625 Hannover, Germany;; ^d^Friedrich-Loeffler-Institut, 17493 Greifswald, Insel Riems, Germany

**Keywords:** porcine SRY gene, sex reversal, CRISPR/Cas9, RNPs, HMG domain

## Abstract

The sex-determining region on the Y chromosome (SRY) is thought to be the central genetic element of male sex development in mammals. Pathogenic modifications within the SRY gene are associated with a male-to-female sex reversal syndrome in humans and other mammalian species, including rabbits and mice. However, the underlying mechanisms are largely unknown. To understand the biological function of the SRY gene, a site-directed mutational analysis is required to investigate associated phenotypic changes at the molecular, cellular, and morphological level. Here, we successfully generated a knockout of the porcine SRY gene by microinjection of two CRISPR-Cas ribonucleoproteins, targeting the centrally located “high mobility group” (HMG), followed by a frameshift mutation of the downstream SRY sequence. This resulted in the development of genetically male (XY) pigs with complete external and internal female genitalia, which, however, were significantly smaller than in 9-mo-old age-matched control females. Quantitative digital PCR analysis revealed a duplication of the SRY locus in Landrace pigs similar to the known palindromic duplication in Duroc breeds. Our study demonstrates the central role of the HMG domain in the SRY gene in male porcine sex determination. This proof-of-principle study could assist in solving the problem of sex preference in agriculture to improve animal welfare. Moreover, it establishes a large animal model that is more comparable to humans with regard to genetics, physiology, and anatomy, which is pivotal for longitudinal studies to unravel mammalian sex determination and relevant for the development of new interventions for human sex development disorders.

## Significance

The present work characterizes the porcine sex-determining region on the Y chromosome (SRY) gene and demonstrates its pivotal role in sex determination. We provide evidence that genetically male pigs with a knockout of the SRY gene undergo sex reversal of the external and internal genitalia. This discovery of SRY as the main switch for sex determination in pigs may provide an alternative for surgical castration in pig production, preventing boar taint. As the pig shares many genetic, physiological, and anatomical similarities with humans, it also provides a suitable large animal model for human sex reversal syndromes, allowing for the development of new interventions for human sex disorders.

In mammalian species, the male and female sex are determined by the presence or absence of the Y chromosome (1). Sex determination is triggered by the expression of specific genes that cause the bipotential gonads to develop into either testes or ovaries (2). The sex-determining region located on the short arm of the Y chromosome (SRY) has been identified as an essential factor for male sex development (3, 4). In pigs, the SRY gene consists of a single exon, with an open reading frame (ORF) of 624 base pairs (bp), which encodes for the 206 amino acids of the testis-determining transcription factor (TDF) (5). Skinner et al. (6) described the porcine SRY gene in Duroc pigs as a palindromic head-to-head duplicated locus, resulting in two SRY loci on the Y chromosome, similar to rabbits (7). Expression of the porcine SRY genes in the male genital ridge can first be detected on day 21 post coitum (p.c.), with highest expression levels between days 21 and 23 p.c. Shortly after the onset of SRY expression (24–27 d p.c.), testis formation can be histologically verified (5, 8). Accordingly, the SRY gene presumably serves as master regulator for the formation of primary precursor cells of the tubuli seminiferi, thus leading to the development of testicles from undifferentiated gonads (9). However, it is still unknown whether the SRY gene is the only sex-determining gene on the Y chromosome or if other genes such as SOX9 (10–12) and SOX3 (13) are also involved in sex determination.

Previously, the SRY gene was knocked out in mice (14) and rabbits (15) by targeting different regions of the gene. Both the knockout (KO) of 92% of the murine SRY gene by transcription activator-like effector nucleases (TALENs) and the CRISPR-Cas–mediated KO of the Sp1–DNA-binding sites in the 5′ flanking region of the rabbit SRY gene resulted in sex reversal. Nevertheless, sequence divergence of the SRY gene between mammalian species limits a direct structural and functional comparison and the investigation of mammalian sex determination. So far, studies investigating the biological function of the SRY gene in mammalian sex determination have only been performed in rodent models, mainly mice. Knowledge about the SRY gene and its biological function in large animal species, especially the domestic pig, is scarce.

The goal of the present study was 1) to evaluate the potential of genome editing to predetermine the sex in livestock species and 2) to establish a large animal model mimicking human sex disorders. Here, we targeted different sites of the porcine SRY gene via intracytoplasmic microinjection of two CRISPR-Cas9 ribonucleoproteins (RNPs) or via cell transfection followed by somatic cell nuclear transfer (SCNT) (*SI Appendix*, Fig. S1). The human and porcine SRY genes are closely related (∼85% amino acid homology) and show similar expression profiles (5, 16). Large animal models are becoming increasingly important in biomedical research due to their great similarities to humans, and the pig is specifically favored (17). The relatively long life expectancy of pigs allows longitudinal studies under conditions that mimic human patients much better than rodent models. A KO of the SRY gene in the porcine model paves the way for a suitable large animal model for the human male-to-female sex reversal syndrome and may offer novel opportunities to address the problem of sex preference for livestock species, which is often associated with animal welfare issues such as surgical castration without anesthesia in the pork industry or culling of male chicks.

## Results

### Duplication of the Porcine SRY Gene.

**Fig. 1.**
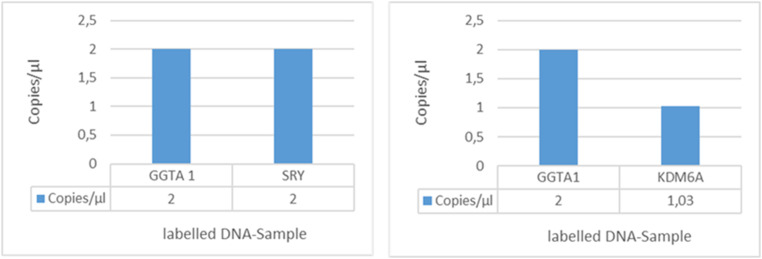
The dPCR biplex assay of WT samples revealed half of the copy number of the monoallelic KDM6A gene compared to the biallelic GGTA1 gene, as expected. A similar copy number of the monoallelic SRY gene compared to the biallelic GGTA1 gene shows a duplication of the SRY locus in Landrace pigs. Reprinted with permission from ref. 50.

Digital PCR (dPCR) (QuantStudio3D, Thermo Fisher Scientific) was employed to check for the SRY gene duplication in wild-type (WT) Landrace pigs. Three targets, including the SRY and the monoallelic KDM6A genes, both located on the Y chromosome, and the biallelic GGTA1 (alpha-1,3-galactosyltransferase) gene located on chromosome 1 were selected for direct comparison of their copy numbers. The copy numbers of GGTA1 were set to two (biallelic), whereas the KDM6A and SRY genes were quantified in relation to the GGTA1 gene. A comparison of the copy numbers of KDM6A and GGTA1 in WT pigs revealed a twofold lower copy number of the monoallelic KDM6A compared to the biallelic GGTA1 in a male WT control, as expected. By contrast, the SRY gene exhibited a similar copy number as the biallelic GGTA1 gene in WT samples (Fig. 1), thereby confirming the duplication of the SRY gene in Landrace pig breeds.

### Investigation of the 5′ Flanking Region of the HMG Domain.

**Fig. 2.**
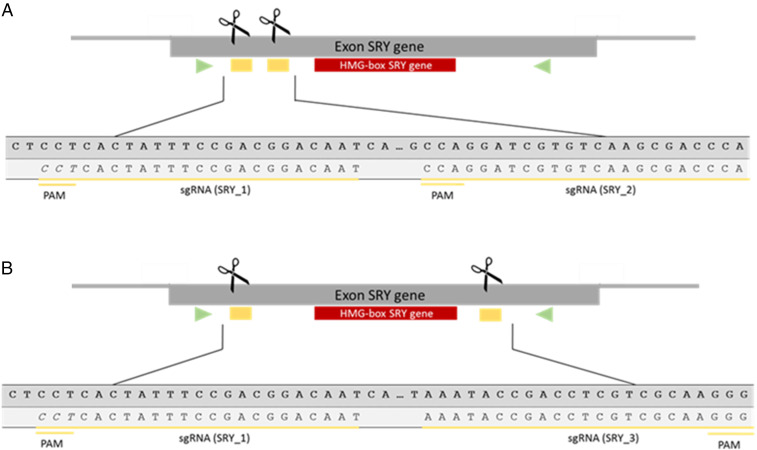
(*A*) Schematic illustration of the gRNAs SRY_1 and SRY_2 (yellow underlined) targeting an ∼72-bp segment in the 5′ flanking region of the HMG domain (red box) of the SRY gene. (*B*) Location of two sgRNA target sites SRY_1 and SRY_3 (yellow underlined) flanking the HMG-box (red box) of the SRY gene. The primers amplifying the SRY exon are indicated with green arrows. Reprinted with permission from ref. 50.

In our first experiment, we introduced an in-frame mutation of −72 bp at the 5′ flanking region of the ″high mobility group″ (HMG) domain of the SRY gene to test whether the 5′ flanking region is essential for SRY function (Fig. 2*A*). After the transfer of 30 embryos generated by intracytoplasmic microinjection of guide RNAs (gRNAs) SRY_1 and SRY_2 and Cas9 protein into each of two recipients, one genetically male piglet (690/1) was born that displayed a male phenotype without sex reversal. Since the −72 bp in-frame mutation did not cause a frameshift within the SRY ORF (*SI Appendix*, Fig. S2), we concluded that the mutation of this part of the 5′ flanking region of the HMG box does not affect formation and function of the SRY protein.

In parallel, male somatic cells were transfected with Cas9 protein and gRNAs SRY_1 and SRY_2. The donor cells were subsequently used to produce two healthy piglets (704/1 and 704/2) by SCNT (Fig. 2*A*) (*SI Appendix*, Fig. S3 and Table S1). PCR and Sanger sequencing of the target site revealed two deletions of 72 bp and 73 bp in each piglet (*SI Appendix*, Figs. S4 and S5). This is consistent with the dPCR results (see *Duplication of the Porcine SRY Gene*) that provided evidence for the presence of two copies of the SRY gene in these Landrace pigs. However, despite the presence of an out-of-frame mutation in the 5′ flanking region of one copy of the SRY gene, both piglets developed a male phenotype and showed no sex reversal. These results further confirmed the duplication of the porcine SRY gene and that expression from one SRY copy is sufficient for the development of male genitalia.

### Production of SRY-KO Pigs.

**Fig. 3.**
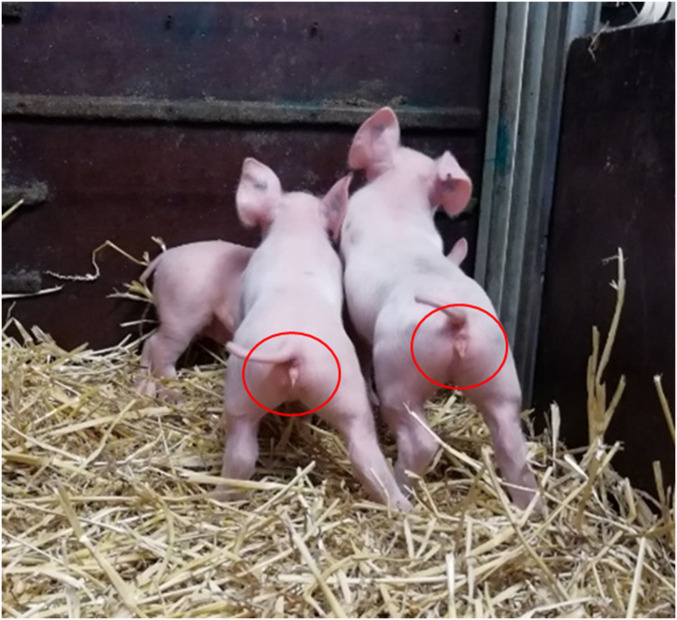
A total of 12 healthy piglets were born after intracytoplasmic microinjection of two CRISPR-Cas9 RNP complexes (SRY_1 and SRY_3) into IVF-produced zygotes and surgical embryo transfer to recipients. Three genetically male piglets (714/1, 715/2, and 715/7) had a complete set of female external genitalia. The deletion of the SRY gene did not affect the growth rate compared to WT controls (*SI Appendix*, Figs. S15 and S16). Reprinted with permission from ref. 50.

**Fig. 4.**
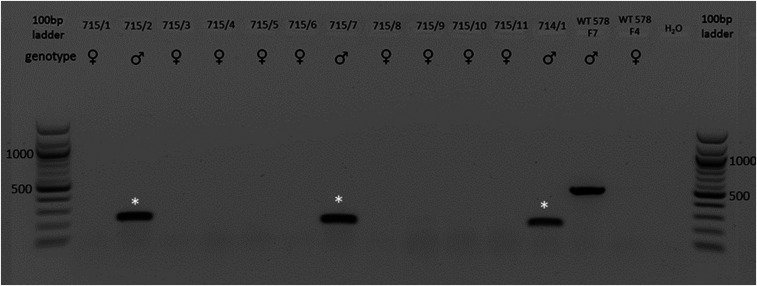
PCR-based detection of the edited SRY gene in piglets (714/1 and 715/1 to 715/11) generated via microinjection of CRISPR-Cas9 RNP complexes (SRY_1 and SRY_3). Three piglets (715/2, 715/7, and 714/1, indicated with white asterisk) showed deletions of ∼300 bp within the SRY gene compared to a male WT control (WT 578 F7). The male WT control showed an expected band of ∼500 bp. A female WT (WT 578 F4) served as negative control. Reprinted with permission from ref. 50.

**Fig. 5.**
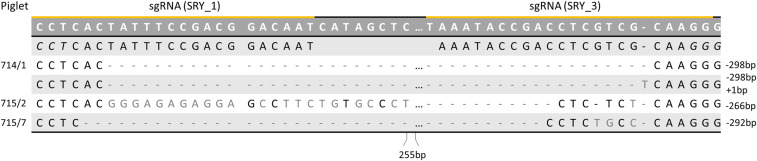
Sanger sequencing of the purified PCR product of the SRY-KO piglets (715/2, 715/7, and 714/1) showed genetic modifications within the SRY locus. Piglet 715/7 displayed a deletion of 292 bp and piglet 715/2 of 266 bp. Piglet 714/1 showed two different modifications with a deletion of 298 bp and an indel formation of −298 bp and +1 bp. Reprinted with permission from ref. 50.

**Fig. 6.**
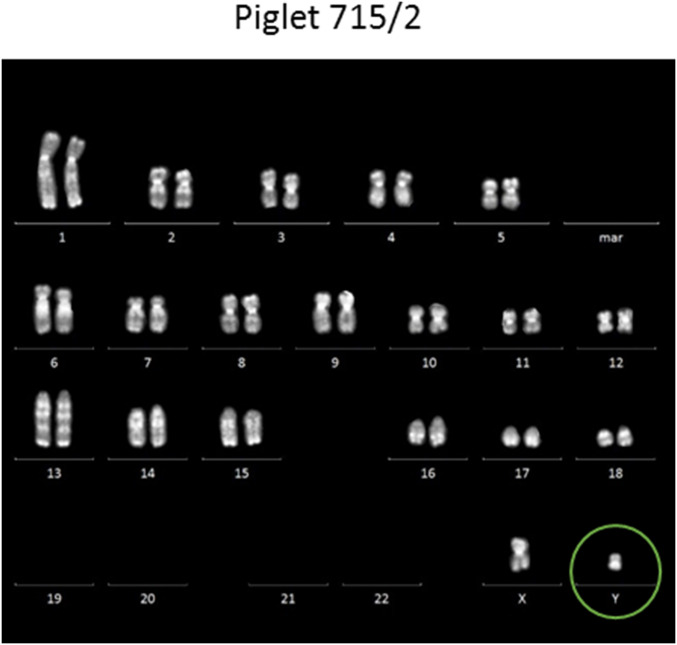
Karyotyping of cells from the SRY-KO piglet 715/2 confirmed the male genotype of this piglet. The karyotypes of piglet 715/7 and 714/1 are shown in *SI Appendix*, Fig. S7.

In the next experiment, we introduced a deletion of ∼300 bp, encompassing the entire HMG domain of the porcine SRY gene (Fig. 2*B*). A total of 31 or 32 embryos derived from intracytoplasmic microinjection of the Cas9 protein and gRNA SRY_1 and SRY_3 into in vitro fertilization (IVF)-produced zygotes were surgically transferred into each of three hormonally synchronized recipient sows. Two recipients went to term and delivered, in total, 12 healthy piglets with a female phenotype (Fig. 3). Three of these piglets (714/1, 715/2, and 715/7) were genetically male and carried a deletion of ∼300 bp encompassing the entire HMG domain of the SRY gene (Table 1 and Fig. 4). Sequencing of the target region revealed frameshift mutations of −266 bp in piglet 715/2 and −292 bp in piglet 715/7. Piglet 714/1 carried two different genetic modifications: a 298-bp deletion and an indel formation consisting of a 298-bp deletion and a 1-bp insertion (Fig. 5). Furthermore, an analysis of six Y-chromosome–specific genes (KDM6A, TXLINGY, DDX3Y, CUL4BY, UBA1Y, and UTY) revealed a male genotype and successful sex reversal in all three piglets (*SI Appendix*, Fig. S6 and Table S2). To ultimately confirm the male genotype of the SRY-KO piglets (715/2, 715/7, and 714/1), cells derived from ear tissue were karyotyped, and the Y chromosome was detected in all three piglets (Fig. 6) (*SI Appendix*, Fig. S7). No chromosomal abnormalities were observed in the sex-reversed pigs 715/2 and 715/7, while piglet 714/1 had an inversion of chromosome 7 (*SI Appendix*, Fig. S7). In silico off-target analysis revealed 34 potential off-target sites within the pig genome (crispor.tefor.net/). We designed primers for the top 10 putative off-target sites for each gRNA (*SI Appendix*, Tables S3 and S4). However, PCR amplification of one off-target site of gRNA_SRY1 and three off-target sites of gRNA_SRY3 remained unsuccessful despite extensive efforts. No off-target events were detected in any of the amplicons (*SI Appendix*, Figs. S8–S10). The recloning of piglet 715/2 led to the birth of seven piglets (735/1 to 735/7) with a sex-reversed phenotype and demonstrated unequivocally that the strategy described in this study could be applied to produce sex-reversed pigs (*SI Appendix*, Figs. S11–S14 and Table S5). All SRY-KO pigs developed normally without any health impairments or apparent deficiencies in weight gain (*SI Appendix*, Figs. S15 and S16 and Tables S6–S8).

**Table 1.** Results of the embryo transfer of microinjected zygotes into recipients

Recipient	Transferred embryos	Pregnancy	Offspring	Genetically male offspring	Genetic modification on the SRY gene	Sex reversal
8018	32	—	—	—	—	—
714	32	+	1	1	1	1 (714/1)
715	31	+	11	2	2	2 (715/2, 715/7)

Overall, 3 (714/1, 715/2, and 715/7) out of 12 piglets showed a sex reversal with a female phenotype and male genotype.

### External and Internal Genitalia of the SRY-KO Pigs.

**Fig. 7.**
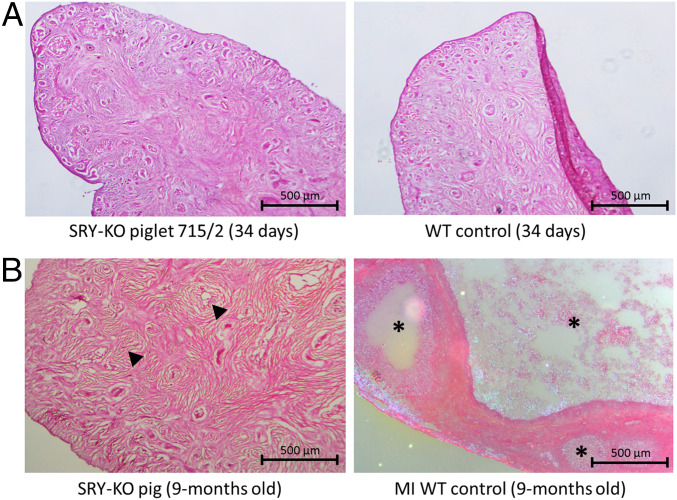
(*A*) Hematoxylin and eosin staining of porcine ovarian tissue from the SRY-KO piglet 715/2 (*Left*) and female WT control (*Right*) 34 d after birth. No structural differences were shown. (*B*) Histological analysis of the ovarian tissue of the SRY-KO pig (*Left*) compared to the female WT control from same litter (microinjection [MI] WT control, *Right*) at the age of 9 mo. A higher amount of loose connective tissue (indicated with black arrows) in the 9-mo-old SRY-KO pig revealed fat deposits within the ovarian tissue. The ovarian tissue from SRY-KO pigs showed no follicular development compared to MI WT controls (black asterisk) at 9 mo of age. (Scale bars, 500 μm.) Reprinted with permission from ref. 50.

We compared the external and internal genitalia of the SRY-KO pigs with age-matched WT females from conventional artificial insemination and unedited female littermates of the SRY-KO pigs produced by microinjection as controls. At the age of 34 d, the external genitalia of the SRY-KO piglets corresponded to the external genitalia of female littermates and WT controls. To investigate the internal genitalia, the ovaries, oviducts, and uteri of the 34-d-old SRY-KO piglets and female controls were prepared. The 34-d-old SRY-KO piglets had complete female internal genitalia, including ovaries, oviducts, and uteri that were similar in size to that of age-matched WT controls (*SI Appendix*, Fig. S17). Moreover, histological analysis of the inner structure of the ovaries revealed no alterations (Fig. 7*A*).

**Fig. 8.**
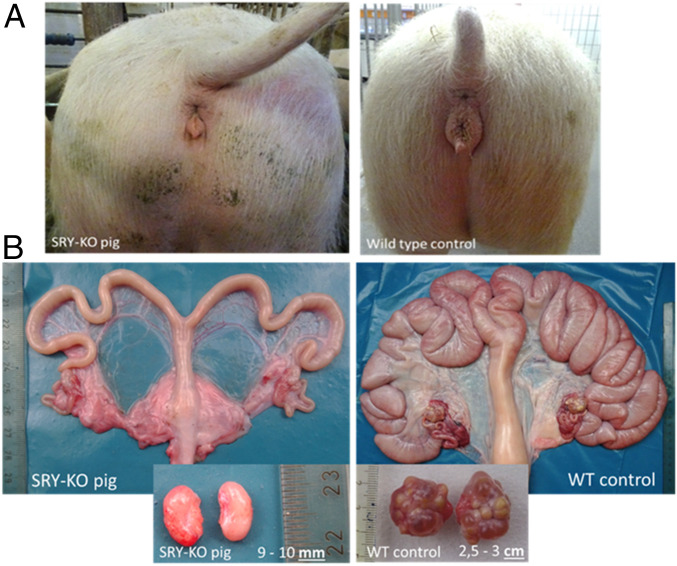
Uteri and ovaries of the 9-mo-old SRY-KO, XY pig (714/1) and the age-matched WT, XX piglet (control from same litter). (*A*) Substantial size differences of external female genitalia were apparent in the 9-mo-old SRY-KO pig compared to the female WT control. (*B*) The ovaries of the 9-mo-old SRY-KO, XY pig were significantly smaller than the ovaries of the WT, XX pig and showed no follicle development.

However, substantial size differences of the female genitalia became obvious in 9-mo-old SRY-KO pigs (Fig. 8). The gene-edited SRY-KO pigs developed a significantly smaller genital tract and were not observed in heat, even after three consecutive treatments of 1,000 IU pregnant mare serum gonadotropin (PMSG) (Pregmagon, IDT Biologika) followed by an intramuscular injection of 500 to 1,000 IU human chorion gonadotropin (hCG) (Ovogest300, MSD Germany) 72 h later to induce estrus. A histological analysis of the ovaries of 9-mo-old SRY-KO pigs revealed a high amount of loose connective tissue and no formation of follicles in contrast to the age-matched WT control (Fig. 7*B*). Overall, no tumorous alterations were found macroscopically and histologically in ovarian tissues.

### Immunohistological Staining of Ovaries from SRY-KO Pigs.

**Fig. 9.**
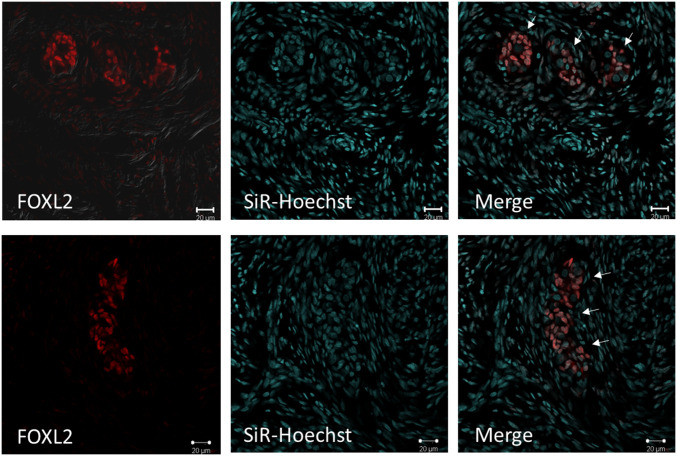
Immunohistological staining of FOXL2-positive cells (red) in ovaries of two 9-mo-old SRY-KO pigs (upper images: SRY-KO pig 1,255, lower images: SRY-KO pig 1,262). Cell clusters of FOXL2-positive cells (indicated with white arrows) were detected in the cortical region of the porcine ovaries. SiR-Hoechst–stained nuclei (blue) are shown. The merged images revealed positive FOXL2 staining in the nuclei of the cells. The experiments were repeated three times with similar results. (Scale bars, 20 μm.) Reprinted with permission from ref. 50.

We further characterized the ovaries from the 9-mo-old SRY-KO pigs by immunohistological staining with the murine oocyte marker forkhead box protein L2 (FOXL2). Staining for FOXL2 revealed several cell clusters mainly located in the cortical regions of the ovaries in SRY-KO pigs. Costaining with carboxylated silicon-rhodamine (SiR-Hoechst) allowed for localization of the positive FOXL2 fluorescence in the cell nucleus (Fig. 9). In the medulla, positive cells were less frequent. In ovaries of the female WT controls, FOXL2-positive cells were sporadically detectable, exhibiting a dispersed cell pattern (*SI Appendix*, Fig. S18). RT-PCR of porcine ovaries revealed a 5.5-fold higher RNA expression of VASA and a 2.5-fold higher OCT4 expression in SRY-KO pigs compared to control females (*SI Appendix*, Fig. S19).

## Discussion

The SRY has so far exclusively been examined in small rodents, where it is critically involved in sex determination (18). However, the role of SRY expression in male sex development in large animals has not been analyzed.

Here, we report the successful KO of the porcine SRY gene by intracytoplasmic microinjection of two CRISPR-Cas9 RNP complexes, which resulted in genetically male pigs with a female phenotype.

The CRISPR-Cas9 system has emerged as the genome-editing technology of choice for targeted genetic modifications due to its ease of use, cost efficiency, and high specificity to introduce mutations at the targeted loci (19, 20). Nevertheless, off-target modifications at undesired genomic sites may occur (21). The use of CRISPR-Cas9 RNPs enables efficient genome editing while significantly reducing possible off-target events and mosaicism (22, 23). In our study, no mosaicism was observed in SRY-KO pigs, which might be explained by the use of CRISPR-Cas RNPs. Moreover, no off-target events were detected at the tested potential off-target sites in the genome of SRY-KO pigs, which was verified by PCR-based analysis and Sanger sequencing. Whole-genome sequencing using accurate and sensitive off-target profiling techniques such as genome-wide, unbiased identification of double-strand breaks enabled by sequencing (GUIDE-Seq) and circularization for in vitro reporting of clevage effects by sequencing (CIRCLE-Seq) could completely exclude the presence of unexpected mutations (21, 24, 25). In one piglet (714/1), an inversion of chromosome 7 was observed. The origin of this clonal cytogenetic aberration remains unclear. However, it is very unlikely that the inversion originated from the use of the CRISPR-Cas system, as no putative off-target site was located on chromosome 7.

A previous study described the porcine SRY gene in Duroc pigs as a palindromic head-to-head duplication of the SRY locus (6), similar to the rabbit SRY gene (7). In our study, a quantitative analysis by dPCR (QuantStudio3D, Thermo Fisher Scientific) confirmed the presence of a duplication of the SRY locus also in the Landrace pig breed by detection of a similar copy number of the SRY and the biallelic GGTA1 gene. Furthermore, the detection of two different deletions within the porcine SRY gene in the piglets produced via SCNT further supports the dPCR results, as the use of SCNT excludes mosaicism in offspring. A previous study in mice indicated the presence of two messenger RNA (mRNA) transcripts (Sry-S and Sry-T) of the SRY gene, where the expression of only Sry-T resulted in complete male-to-female sex reversal (26). Whether this also applies to pigs needs to be investigated in future studies. Yet, our results revealed that one copy of the porcine SRY gene is sufficient for male genitalia development.

KO of the entire HMG domain followed by a frameshift mutation of the downstream sequence resulted in healthy, genotypically sex-reversed males that showed normal development and growth rates. However, substantial size differences of all female genitalia became obvious in 9-mo-old SRY-KO pigs compared to age-matched WT controls, thus demonstrating a markedly retarded development. It is unclear whether Y-chromosomal gene and hormone expression hampered the development of female genitalia in SRY-KO pigs. A prominent example of the influence on female sex development of perturbed hormone profiles (androstenone and müllerian inhibition substance [MIS]) is the bovine freemartin syndrome, which leads to masculinization of the female genitalia (27). Moreover, persistent expression of the MIS in female mice resulted in cord-like ovaries, which are depleted of germ cells (28). The absence of a second X chromosome in the SRY-KO pigs might have disturbed regular female sex development, since the inactivation of one copy of the X chromosome is essential for undisturbed female development (29). Nevertheless, expression profiling of the human X chromosome revealed that 34 of 224 transcripts (genes especially located on the short arm of the X chromosome) escape X chromosome inactivation (30). Presumably, expression of specific genes on the second X chromosome is required for primordial germ cells to advance to mature follicles and to prevent stromal fibrosis of the ovaries (31). This implies that certain expression levels of X-linked genes from both X chromosomes trigger and influence female sex maturation. The generation of a Y chromosome KO resulting in an X0 genotype would be a promising animal model to further unravel the influence of Y-chromosomal gene expression and the biological importance of the second X chromosome in female sex development. CRISPR-Cas–mediated elimination of the murine Y chromosome by targeting a cluster of genes along the Y chromosome has previously been shown (32). This may also be a feasible option to generate a porcine X0 genotype.

A progressive loss of primordial germ cells at the early stages in ovarian development in humans leads to the formation of wavy connective tissue, so called “streak gonads” (31, 33). Immunohistological staining of SRY-KO ovaries for FOXL2, a marker for ovarian differentiation (34), revealed positively stained cells clustered in the cortical regions. We conclude that these clustered cells are precursors of the porcine oocytes that show the ability to form follicle-like structures that fail to further differentiate into mature follicles. FOXL2-positive cells were found in ovaries of SRY-KO pigs, albeit less frequently and in a more dispersed cell pattern than in female WT controls. These cells are often found in the early stages of follicular development and only sporadically observed in the ovaries of mature sows. To verify if the FOXL2-positive cells in porcine ovaries are indeed precursor cells of oocytes, additional markers such as OCT4 (35), VASA (36), DAZL (37), and MIK67 (38) can be used for costaining. Unfortunately, the tested antibodies for VASA and OCT4 did not give specific signals in the immunohistological staining. Therefore, we performed RT-PCR and revealed an ∼5.5-fold higher RNA expression of VASA and 2.5-fold higher expression of OCT4 in 9-mo-old SRY-KO pigs compared to female WT controls. Further studies are required to assess their potential as predictive cellular markers in porcine oocyte differentiation to better characterize FOXL2-positive cells.

Disorders of sex development are defined as congenital conditions with complete failure or rudimentary development of anatomical and chromosomal sex. Most cases of human male-to-female sex reversal syndrome (Swyer syndrome) are associated with mutations in or dysfunctions of the SRY gene which are mainly located within the HMG domain (39, 40). Humans with Swyer syndrome display gonadal dysgenesis characterized by streak gonads and infertility, which is comparable to our findings in SRY-KO pigs. Whereas the HMG domain of the murine SRY gene only shows 75% similarity to the human SRY gene, the porcine and human SRY genes are more closely related (∼85% amino acid homology). The N-terminal (NTD) and C-terminal (CDT) domains of the SRY gene share much less sequence homology as the highly conserved HMG domain of the SRY gene. Nevertheless, both domains of pig and human (∼46% of the NTD domain and ∼37% of CDT domain) are more closely related than mouse and rabbit (5, 16). Taking this into account, the high sequence similarity, the similar expression profiles of the SRY gene, and the high degree of physiological, genetic, and anatomical similarity between pigs and humans render the pig a promising large animal model for human disorders in sex development, especially the Swyer syndrome (5, 39, 41).

This study paves the way for using this approach to predetermine the sex in pigs, which would be of great benefit for animal welfare by eliminating the need to castrate male offspring to avoid boar taint. Currently, most piglets are surgically castrated without anesthesia shortly after birth, which raised animal welfare concerns and resulted in a ban of this practice in several European countries. Nevertheless, castration without anesthesia is still practiced widely. It was recently reported that the KO of the KISSR gene by TALEN-mediated mutagenesis resulted in the generation of boars that remained in the prepubertal stage and had no boar taint (42). However, for breeding, hormonal treatment is required, which might result in a decreased consumer acceptance of pork. Our results offer a way by using genome editing to influence sex selection. By integrating a spermatogenesis-specific CRISPR-Cas9 vector targeting the HMG domain of the SRY gene into the porcine genome, boars could be generated that produce only phenotypically female offspring. Alternatively, the CRISPR-Cas vector could target multiple genes on the Y chromosome during spermatogenesis to prevent the development of Y-chromosomal sperm. Thereby, only genetically and phenotypically female offspring would be generated. In both approaches, employing a self-excising vector would result in the generation of nontransgenic offspring. Due to a limited number of available SRY-KO pigs, only preliminary studies on the growth performance could be performed in this study. Further studies concerning the growth performance of the SRY-KO pigs are likely needed if this technology is ever to be applied for production purposes. Nevertheless, the preliminary results do not indicate a negative effect of the SRY KO on the growth performance. Whether products from genome-edited animals will find market acceptance in light of a controversial public debate on genome engineering in many countries remains to be seen. At present, genetically modified food-producing animals have already entered the market. The most prominent example is genetically engineered Atlantic salmon in Canada and the United States (43). It is not yet possible to assess how genome editing regulations, especially those using the CRISPR-Cas system, will evolve. Overall, genome editing might improve animal welfare in pig farming and lead to a more sustainable pork production process.

## Materials and Methods

### Animal Welfare.

Animals were maintained and handled according to the German guidelines for animal welfare and the genetically modified organisms act. The animal experiments were approved by an external animal welfare committee (Niedersächsisches Landesamt für Verbraucherschutz und Lebensmittelsicherheit [LAVES] file no. 33.9-42502-04-17/2541), which included ethical approval of the experiments.

### Transfection of gRNAs.

The CRISPR-Cas9 system was used to induce defined deletions within the SRY gene (Ensembl transcript: ENSSSCG00000037443). gRNAs targeting either the 5′ flanking region of the HMG domain of the SRY gene (SRY_1 and SRY_2) or encompassing the HMG box (SRY_1 and SRY_3) were designed using the web-based design tool *CRISPOR* (http://crispor.tefor.net/) (Fig. 2). Target sequences were analyzed via BLAST to reduce the probability for off-target events. The gRNA oligos with a BbsI overhang were cloned into the linearized CRISPR-Cas9 vector pX330 (Addgene, 42230). Afterward, two CRISPR-Cas9 plasmids were cotransfected (at a final concentration of 5 μg/μL) into male porcine fibroblasts by electroporation (Neon Transfection System, Thermo Fisher Scientific) to test the efficacy of the plasmids to induce double-strand breaks at the targeted locus. Electroporation conditions were as follows: 1,350 V, 20 mm, and two pulses. After lysis of transfected cells, the cell lysate was analyzed using SRY-specific primer (SRY-F: 5′-TGA​AAG​CGG​ACG​ATT​ACA​GC and SRY-R: 5′-GGC​TTT​CTG​TTC​CTG​AGC​AC-3′). The purified PCR product (10 ng/μL) (Invisorb Fragment CleanUp, Startec) was Sanger sequenced to detect mutations at the target site.

### IVF and In Vitro Maturation.

In vitro maturation of porcine oocytes was performed as previously described (44). Briefly, porcine oocytes were collected from ovaries derived from slaughterhouse and matured for 40 h in a chemically defined maturation medium supplemented with three cytokines (FGF2, LIF, and IGF1) in combination, the so-called “FLI medium.” For IVF, frozen boar semen from a fertile Landrace boar was thawed for 30 s in a water bath (37 °C). The motility of sperm was analyzed using microscopy (Olympus, BH-2). After washing with Androhep Plus (Minitube) and centrifugation for 6 min at 600 × *g*, ∼75 to 100 sperm per oocyte (depending on semen capacity) were used for fertilization (no sexed sperm were utilized for fertilization). After 4 h of coincubation, the fertilized oocytes were cultured in porcine zygote medium (PZM-3 medium).

### SCNT.

SCNT was performed as previously described (45). Fetal fibroblasts transfected with Cas9 protein and gRNA SRY_1 and SRY_2 targeting the flanking region of the HMG domain of the SRY gene were used as donor cells. A total of 82 and 86 one- to two-cell embryos were surgically transferred into two hormonally synchronized German Landrace gilts (7 to 9 mo old). Estrus had been synchronized by application of 20 mg/d/gilt Altrenogest (Regumate 4 mg/mL, MSD Germany) for 12 d, followed by an injection of 1,000 IU PMSG (Pregmagon, IDT Biologika) on day 13 and induction of ovulation by intramuscular injection of 500 to 1,000 IU hCG (Ovogest300, MSD Germany) 72 h after PMSG administration.

### Preparation of RNP Complexes for Microinjection.

The Alt-R CRISPR-Cas9 system (IDT) consists of two CRISPR RNA components (crRNA and tracrRNA). The crRNA was individually designed to target the HMG domain of the SRY gene (SRY_3: 5′-AAA​TAC​CGA​CCT​CGT​CGC​AA-3′). To generate an active gRNA, both components (crRNA and tracRNA) were annealed at 95 °C for 5 min and then ramped down to 25 °C at 5 °C/min in a ratio of 1:1 to reach a final concentration of 1 µg/µL. Afterward, the gRNA complex was mixed with Alt-R S.p. Cas9 nuclease 3NLS and incubated for 10 min at room temperature to form an active RNP complex at a final concentration of 20 ng/µL. The second RNP complex was prepared using the individually designed synthetic single-guide RNA (sgRNA) (SRY_1: 5′-ATT​GTC​CGT​CGG​AAA​TAG​TG-3′) from Synthego. The sgRNA was mixed with purified 2NLS-Cas9 nuclease using a ratio of ∼1:1.5 (0.84 µL sgRNA [25 pmol] and 1.25 µL Cas9 protein [25 pmols]) and incubated for 10 min at room temperature. After centrifugation at 10,000 rpm for 10 min and 4 °C, the supernatant was transferred to a new tube. Both RNP complexes were mixed in a ratio of 1 (SRY_1) to 1.7 (SRY_3) in microinjection buffer (10 mM Tris, 0.125 mM ethylenediaminetetraacetic acid [EDTA]) and directly used for microinjection.

### Microinjection.

The RNPs (SRY_1 and SRY_3) targeting the entire HMG domain of the SRY gene were intracytoplasmatically coinjected into IVF-produced zygotes derived from oocytes collected from slaughterhouse ovaries 20 h after fertilization. To this end, ∼10 pl of the RNP solution was injected with a pressure of 600 hPa into IVF-produced zygotes (FemtoJet, Eppendorf). The injected zygotes were cultured in PZM-3 medium at 39 °C, 5% CO_2_, and 5% O_2_. At day 5, when embryos had reached the blastocyst stage, 31 or 32 embryos, respectively, were surgically transferred into recipients.

### Establishing Cell Cultures from SRY-KO Piglets.

Porcine fibroblasts were isolated from ear tissue of the piglets and cultured in Dulbecco’s modified Eagle’s medium with 2% penicillin/streptomycin, 1% nonessential amino acids and sodium pyruvate, and 30% fetal calf serum (Gibco, 10270-106). When cells reached confluency, they were lysed with tail lysis buffer, and genomic DNA was analyzed by PCR and karyotyping.

### PCR-Based Genotyping.

Genomic DNA of the pigs was extracted from tail tips. Cells were isolated from ear tissue. The DNA concentration was determined using the NanoDrop (Thermo Scientific) system. For genotyping of the pigs, PCR was employed using specific primer (SRY-F: 5′-TGA​AAG​CGG​ACG​ATT​ACA​GC-3′ and SRY-R: 5′-GGC​TTT​CTG​TTC​CTG​AGC​AC-3′) flanking a 498-bp segment of the SRY gene (Fig. 2*B*). PCR amplification was performed in a total volume of 50 µL: 20 ng DNA, 0.6 µM reverse and forward primer, 1.5 mM MgCl_2_, 0.2 mM dNTPs, and 1.25 U Taq Polymerase. Cycling conditions were as follows: 32 cycles with denaturation at 94 °C for 30 s, annealing at 59 °C for 45 s, extension at 72 °C for 30 s, and a final extension at 72 °C for 5 min. The standard conditions for gel electrophoresis were set up to 80 V, 400 mA, and 60 min using a 1% agarose gel. The PCR product was purified (InvisorbFragment CleanUp-Kit, Startec) and Sanger sequenced. To further analyze the genotype of the piglets, Y-chromosome–specific genes such as KDM6A, DDX3Y, CUL4BY, UTY, UBA1Y, and TXLINGY were amplified (*SI Appendix*, Table S2).

### Karyotyping of the Cells.

Karyotyping was performed on porcine fibroblasts isolated from ear tissue. After treatment of cells for 30 min with colcemide (Invitrogen), cells were trypsinized and metaphase chromosomes were prepared according to standard procedures. Fluorescence R-banding using chromomycin A3 and methyl green was performed as previously described (46). At least 15 metaphases were analyzed per offspring. The standard karyotype of the pig includes 38 chromosomes. Karyotypes were described according to Gustavsson (47) and the International System for Human Cytogenetic Nomenclature.

### Histology and Immunohistological Staining.

Porcine ovarian tissues were fixed with 4% paraformaldehyde for 6 to 8 h (smaller tissues of up to 5 × 10 mm) or overnight (tissues of up to 2 × 3 cm) and subsequently incubated in 30% sucrose for 2 h and frozen at −80 °C. Afterward, the tissues were embedded in TissueTek (Sakura, TTEK) and cut into thin sections (25 μm). Sections were stained with hematoxylin and eosin following standard procedures (48), and the inner structure of ovaries was analyzed by microscopy (DMIL LED, Leica). For immunohistological staining, ovarian slides were washed three times in washing solution (0.02 M phosphate-buffered saline [PBS] with 0.1% Triton X-100) for 15 min at room temperature. The plasma membrane was permeabilized with 0.5% Triton X-100 (dissolved in 0.02 M PBS) for 30 min at room temperature. Afterward, samples were blocked with 2% bovine serum albumin and 2% horse serum to prevent nonspecific binding of the secondary antibody to antigens in ovarian tissue. After another washing step, the unlabeled primary antibody FOXL2 (1:150, ab246511, Abcam) or the rabbit IgG isotype (1:150, stock solution: 200 μg/0.5 mL) for control were dripped on ovarian tissue and incubated for 40 h at 4 °C in a humidified chamber. After incubation with the primary antibody, slides were washed three times and stained with fluorescence-labeled secondary antibody AlexaFluor555 donkey anti-rabbit (1:1,000, A31572, Invitrogen) for 90 min at room temperature in a humidified chamber. The ovarian tissue was counterstained with 0.1 mM SiRHoechst solution (SC007: SiR-DNA Kit, Spinochrome) diluted in 0.02 M PBS (1:600) overnight. Tissue sections were covered with 10 μL mounting medium (Vectashield, ZG0326, Vector Laboratories) and a coverslip and dried for at least 2 h until microscopically imaging (stereoconfocal microscopy). Both controls (isotype control and negative control for second antibody) were used for standardization of confocal microscopy parameters.

### RT-PCR.

For RT-PCR, mRNA was isolated from ovarian tissue using Dynabeads mRNA Direct Kit (Life Technologies), and poly(A)-RNA was enriched by NucleoTrapmRNA Kit (MACHEREY-NAGEL). After RNA isolation, 15 ng of RNA was mixed with 4 μL MgCl_2_ (25 mM), 2 μL PCR buffer (10×), 2 μL dNTPs (10 mM), 1 μL hexamers, 1 μL RNase inhibitor (20 U/μL), and 1 μL murine leukemia virus reverse transcriptase (50 U/μL) for complementary DNA (cDNA) amplification under following cycler conditions: priming of hexamers at 25 °C for 10 min, reverse transcriptase at 42 °C for 60 min, and denaturation at 99 °C for 5 min. For RT-PCR, 2 μL cDNA was mixed with 0.4 μL of the upper and lower primer (5 μM) (*SI Appendix*, Table S9) and 10 μL of supplied Power SYBR Green PCR Master mix. PCR cycler conditions were set as follows: heat inactivation of Taq polymerase for 10 min at 95 °C, followed by 40 cycles of 95 °C for 15 s and 60 °C for 1 min. The results from RT-PCR were analyzed using a 7500 Fast Real-Time PCR System (version 1.5.1, Applied Biosystems). For relative quantitation, the reference genes GAPDH and EEF1A1 were used as internal controls.

### Off-Target Analysis.

The top 10 off-target effects were selected from the gRNA design tool *CRISPOR* (crispor.tefor.net/). The PCR primers used for amplifying the PCR product are listed in the *SI Appendix* (*SI Appendix*, Table S3 for SRY_1 and *SI Appendix*, Table S4 for SRY_3). The PCR product was purified (Invisorb Fragment CleanUp-Kit, Startec, Germany) and analyzed via Sanger sequencing.

### dPCR.

Three assays including a probe and two primers (in a ratio of 2.5 probe to 9 nM primer) targeting the SRY and KDM6A genes (fluorescent dye-labeled) on the Y chromosome and GGTA1 gene (hexachlorofluorescein [HEX]-labeled) on chromosome 1 (as control) were designed (*SI Appendix*, Table S10) from IDT for dPCR. The dPCR was performed in a total reaction volume of 14.5 μL with the following components: 7.3 μL Master Mix (QuantStudio3D Digital PCR Master Mix v2, Thermo Fisher Scientific), 0.7 μL HEX and VIC dye-labeled assays each, 1.4 μL diluted genomic DNA, and 4.4 μL nuclease-free water. Standard dPCR thermal cycling conditions were used with an annealing temperature of 60 °C in the QuantStudio 3D Digital device (Thermo Fisher Scientific). Copy numbers of the genes within each chip were compared via the QuantStudio 3D AnalysisSuite software (http://apps.lifetechnologies.com/quantstudio3d/). The copy number of the GGTA1 gene was set at two (biallelic), and the copy numbers of KDM6A and SRY genes were given in proportion to the GGTA1 gene. All findings were verified in three replicates with variable DNA concentration and different WT samples (49).

## Supplementary Material

Supplementary File

## Data Availability

All study data are included in the article and *SI Appendix*.
