# Supplementary material for: Knockout of the HMG domain of the porcine SRY gene causes sex reversal in gene-edited pigs
Source: Proc Natl Acad Sci U S A. 2020 Dec 22;118(2):e2008743118. doi: 10.1073/pnas.2008743118 (PMC7812820; doi:10.1073/pnas.2008743118)
Supplement: Supplementary File [file pnas.2008743118.sapp.pdf]

PNAS
www.pnas.org

Supplementary Information for

Knockout of the HMG domain of the porcine SRY gene causes sex reversal in gene-edited pigs

Stefanie Kurtz[1], Andrea Lucas-Hahn[1], Brigitte Schlegelberger[2], Gudrun Göhring[2], Heiner Niemann[3], Thomas C. Mettenleiter[4], Björn Petersen[1*]

[1]Institute of Farm Animal Genetics, Friedrich-Loeffler-Institut, Mariensee, 31535 Neustadt am Rübenberge, Germany
[2]Institute of Human Genetics, Hannover Medical School, 30625 Hannover, Germany
[3]Clinic for Gastroenterology, Hepatology and Endocrinology, Hannover Medical School, 30625 Hannover, Germany
[4]Friedrich-Loeffler-Institut, 17493 Greifswald, Insel Riems, Germany

*Corresponding author: bjoern.petersen@fli.de; +49 5034 8715196; Institute of Farm Animal Genetics, Friedrich-Loeffler-Institut, Höltystraße 10, 31535 Neustadt am Rübenberge, Germany

This PDF file includes:

Figures S1 to S19

Tables S1 to S10

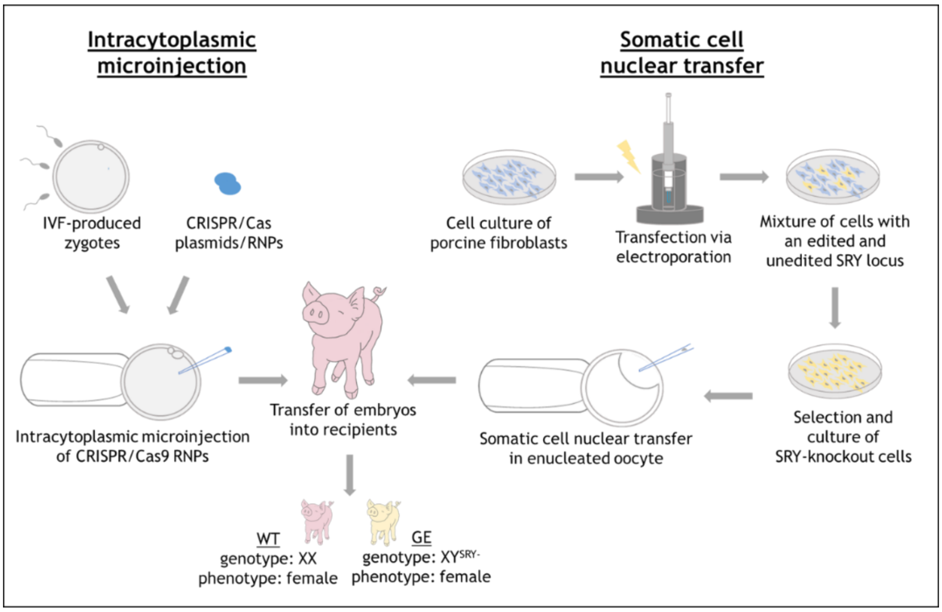

Figure S1 Schematic illustration of the experimental design to generate SRY-KO pigs (XY^{SRY-}) by either intracytoplasmic microinjection of two CRISPR/Cas9 plasmids or RNP complexes into IVF-produced zygotes or somatic cell nuclear transfer (SCNT) of edited cells. Embryos were surgically transferred into hormonally synchronized recipients, and the offspring were analyzed pheno- and genotypically.

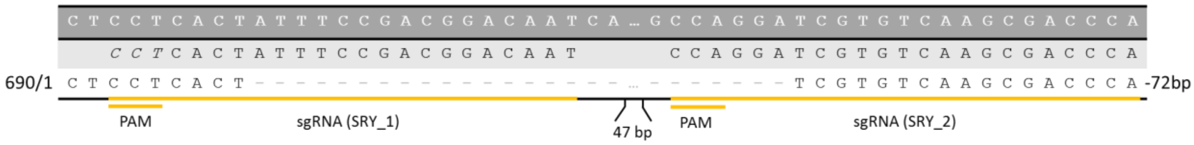

Figure S2 Sanger sequencing of piglet 690/1 revealed a 72 bp in-frame mutation within the 5' flanking region of the HMG domain of the SRY gene after intracytoplasmic microinjection of two CRISPR/Cas plasmids (SRY_1 and SRY_2).

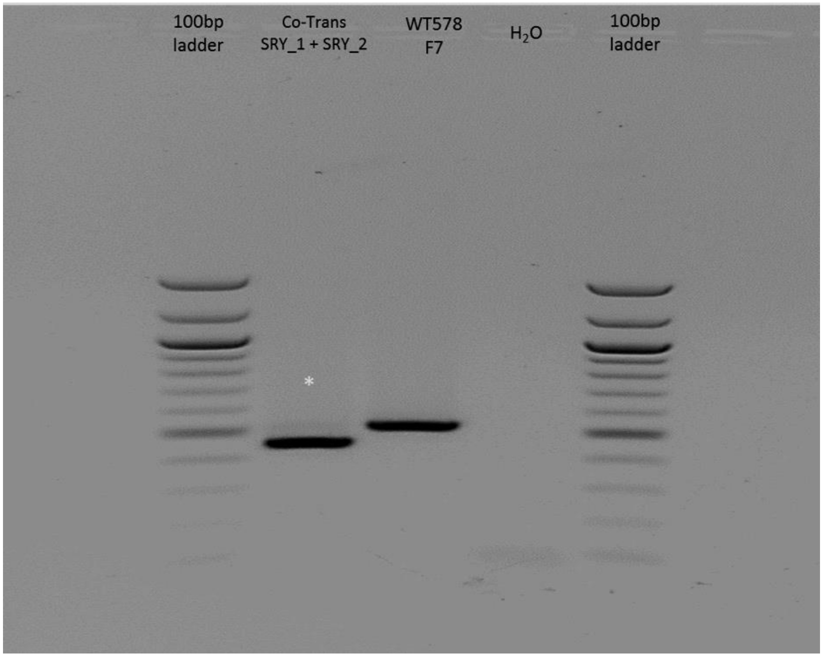

Figure S3 PCR after co-transfection of two plasmids (SRY_1 and SRY_2) in male fetal fibroblasts and selection of the edited cells via single cell dilution (lower band). The edited cells revealed a mutation of approx. 70bp (white asterisk) compared to WT control. The male WT control (WT 578 F7) showed an expected band of approx. 500bp. The edited cells were further employed for somatic cell nuclear transfer.

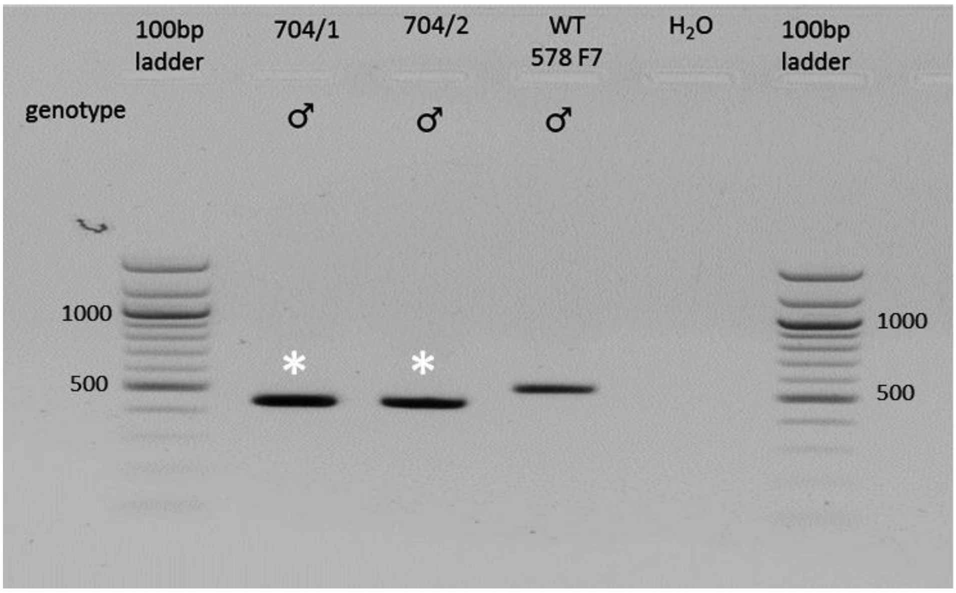

Figure S4 PCR to detect genetic modifications in the SRY gene of the two piglets (704/1-2) generated via SCNT using cells edited with gRNA SRY_1 and SRY_2. Both piglets showed a mutation of approx. 70bp compared to the male WT control (WT 578 F7). The WT control gave the expected band of ~500bp.

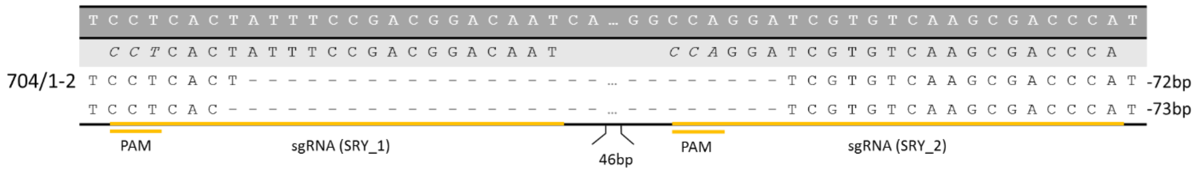

Figure S5 Both piglets (704/1 and 704/2) generated via SCNT using donor cells targeting the 5’ flanking region of the HMG box of the SRY gene showed two different modifications including a deletion of 72bp (in-frame mutation) and 73bp (out-of-frame mutation).

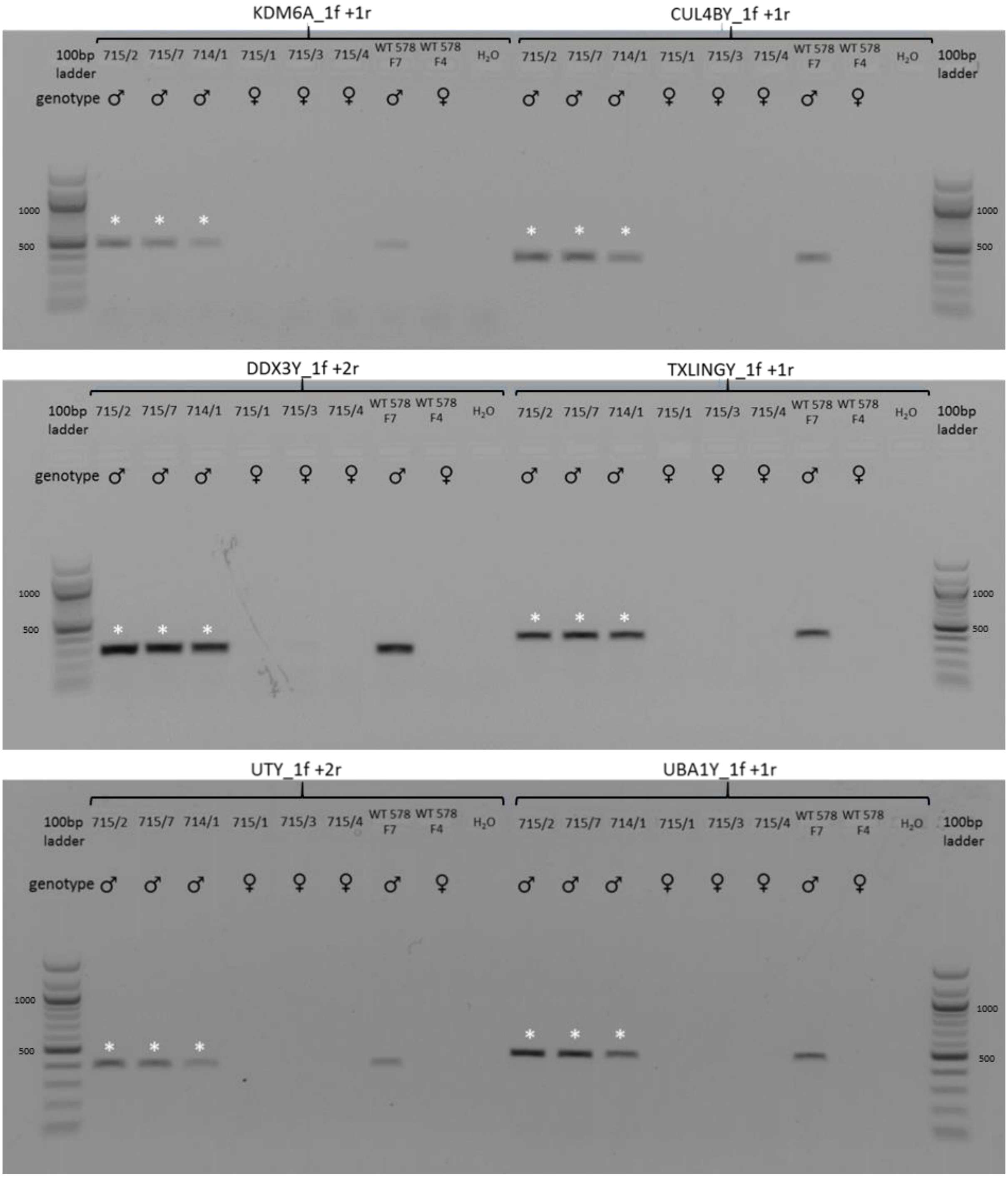

Figure S6 PCR of six different Y chromosome specific genes (KDM6A, CUL4BY, DDX3Y, TXLINGY, UTY and UBA1Y) for detection of the Y chromosome in SRY-KO piglets (715/2, 715/7 and 714/1, indicated by a white asterisk) compared to female wild type controls (715/1, 715/3 and 715/4) from same litter. Moreover, a male (WT 578/F7) and a female (WT 578/F4) DNA sample were used as positive and negative control.

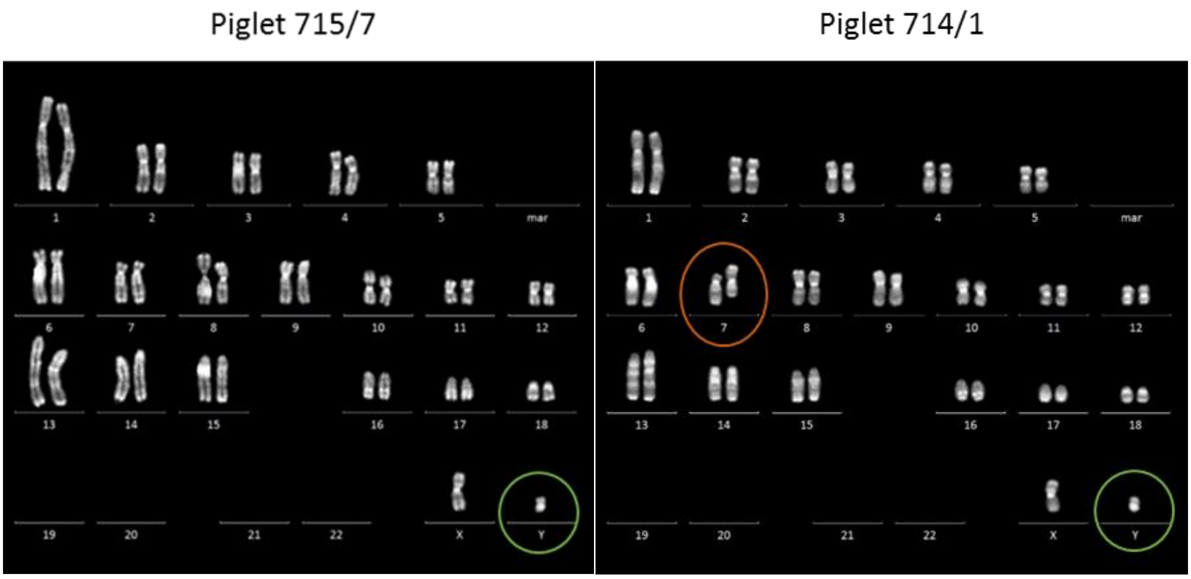

Figure S7 Karyotyping of the SRY-KO piglet 715/7 and 714/1 confirming the male genotype by analysis of the sex chromosomes. In piglet 714/1, a clonal aberration (inversion) was found on chromosome 7.

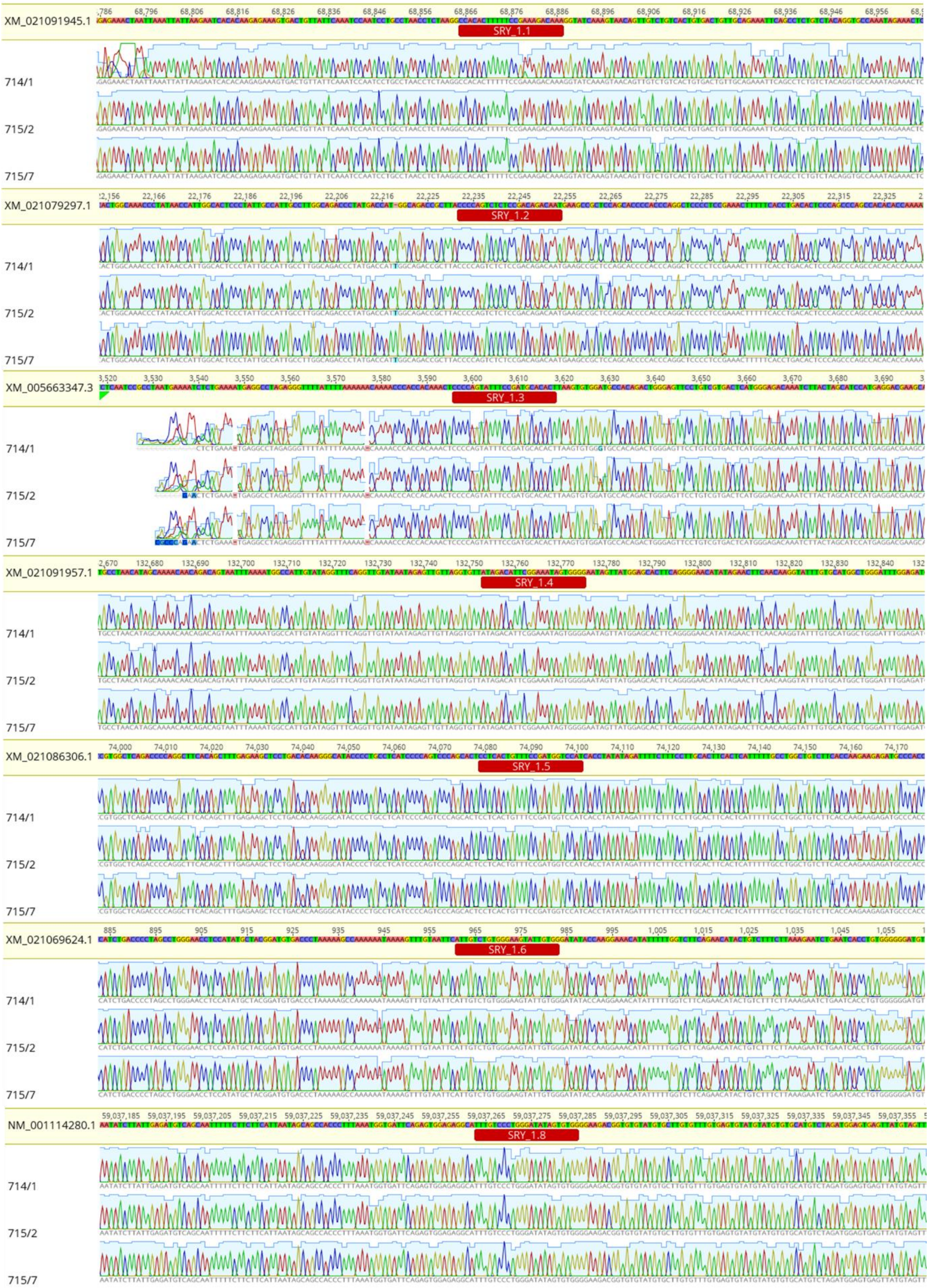

Figure S8 Sanger Sequencing for detecting potential off-target sites of gRNA SRY_1 in SRY-KO pigs 714/1, 715/2 and 715/7. Overall, no off-target events were found.

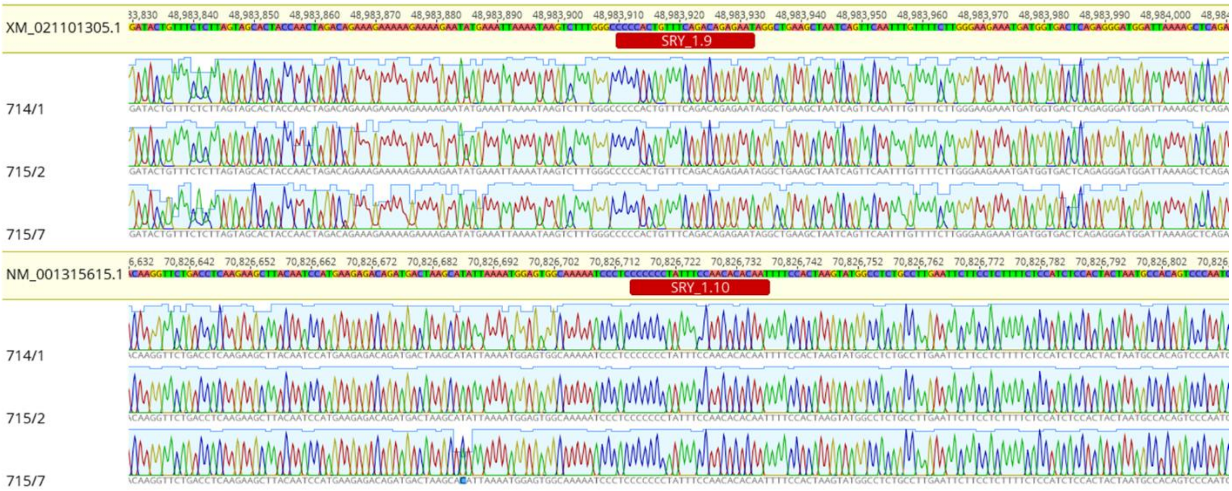

Figure S9 Sanger Sequencing for detecting potential off-target sites of gRNA SRY_1 in SRY-KO pigs 714/1, 715/2 and 715/7. Overall, no off-target events were found.

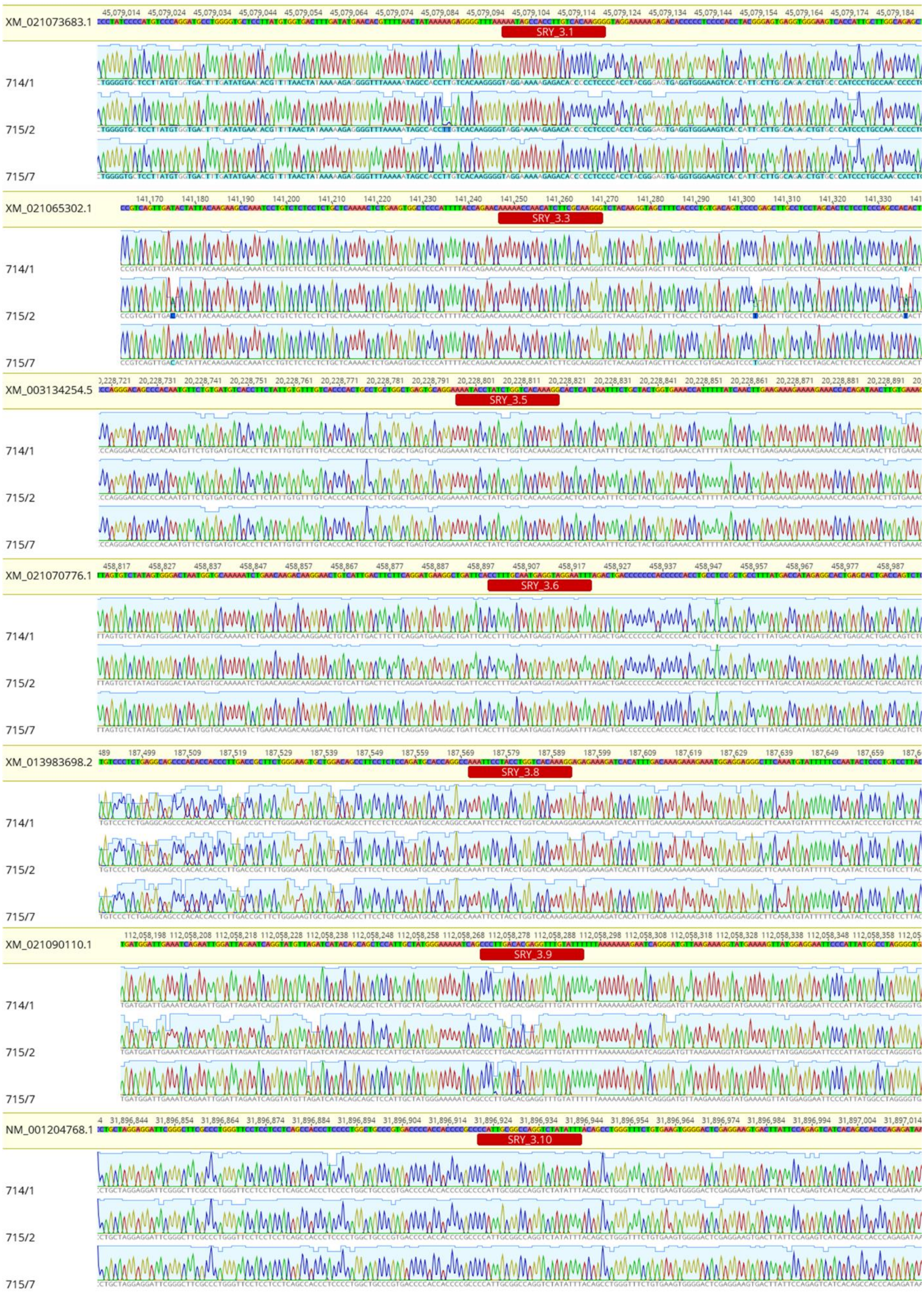

Figure S10 Sanger Sequencing for detecting potential off-target sites of gRNA SRY_3 in SRY-KO pigs 714/1, 715/2 and 715/7. No off-target events were found.

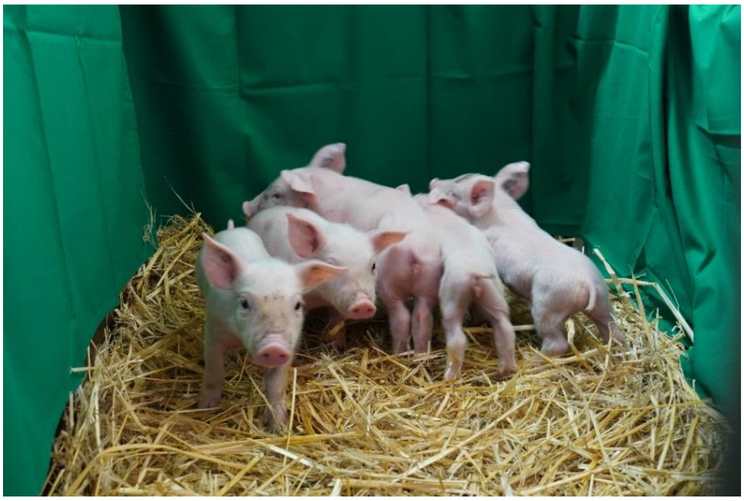

Figure S11 Healthy piglets with a female phenotype and male genotype were born from re-cloning of cells from the SRY-KO piglet 715/2.

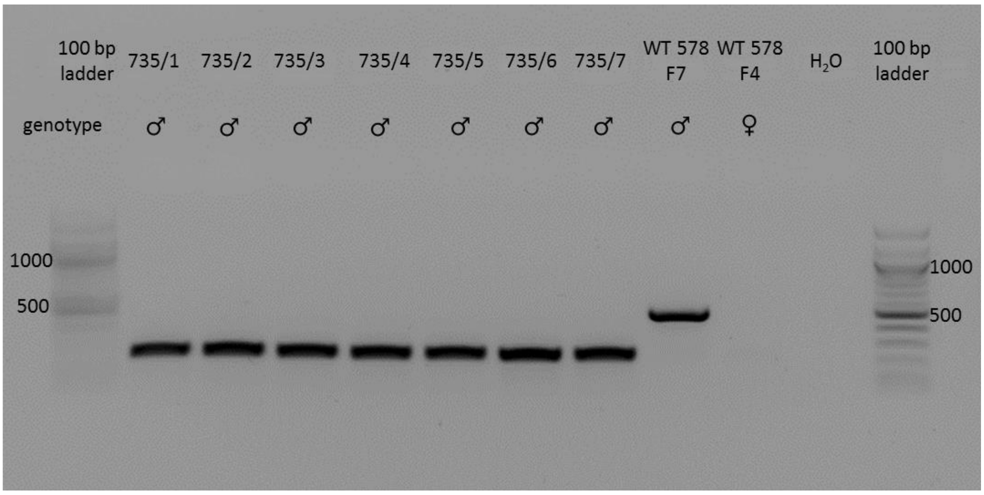

Figure S12 PCR of piglets (735/1 to 7) from re-cloning of cells from the SRY-KO piglet 715/2. All piglets (735/1 to 7) showed deletions of ~300 bp within the SRY gene in contrast to the expected band of approx. 500bp in a male wild type control (WT 578 F7). WT 578 F4 was used as female WT control.

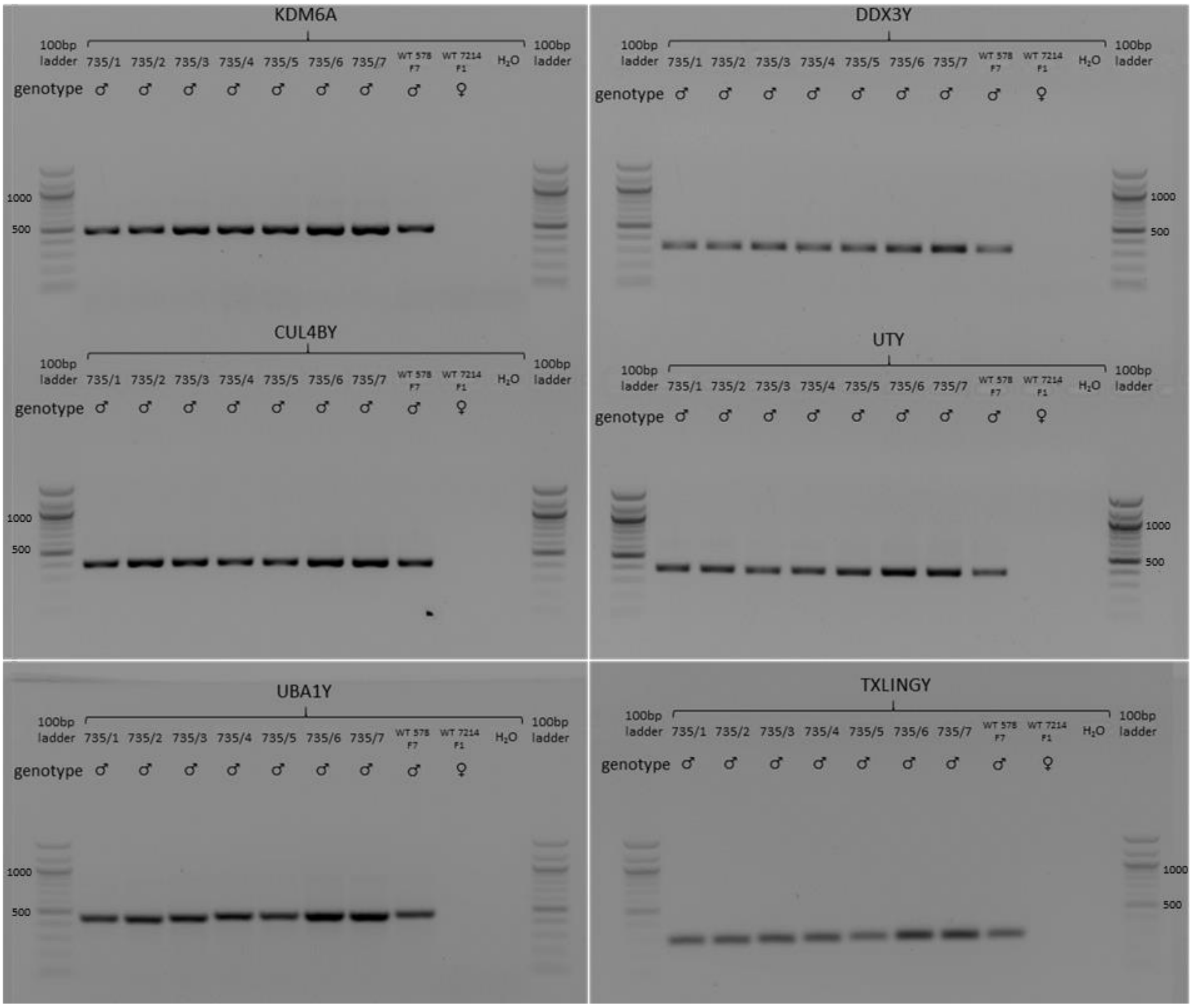

Figure S13 Detection of six different Y chromosome specific genes (KDM6A, CUL4BY, DDX3Y, TXLINGY, UTY and UBA1Y) via PCR in SRY-KO piglets from re-cloning (735/1 to 7). Male (WT 578/F7) and female (WT 7214/F1) DNA samples were used as positive and negative control.

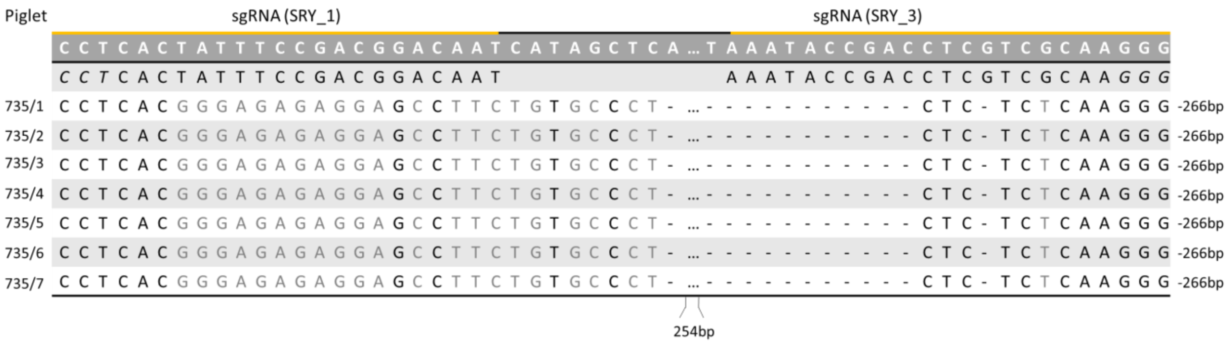

Figure S14 Sanger sequencing revealed a deletion of 266bp in all of the piglets (735/1 to 7) generated via re-cloning of the SRY-KO piglet 715/2.

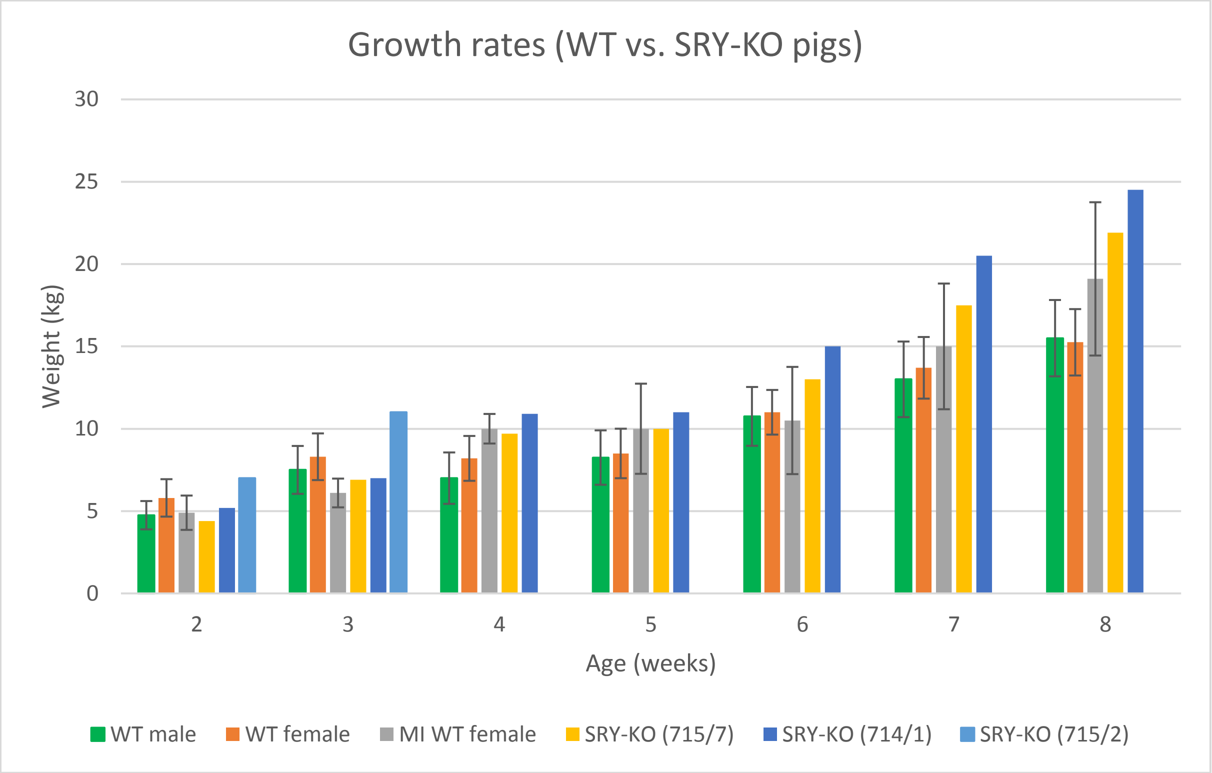

Figure S15 Schematic diagram displaying the weight development (median of growth rates) of the SRY-KO pigs 715/2 (light blue), 715/7 (yellow) and 714/1 (dark blue) compared to the male (green) and female WT controls (orange) and female littermates (MI WT females, grey) with the age of 2 to 8 weeks. The weights of pigs were determined once per week and the median of growth rates per group was calculated to illustrate the diagram. Growth weight of piglet 715/2 is shown until week three, as the pig was sacrificed on day 34 after birth to investigate the internal female genitalia. WT males (n)= 6; WT females (n)= 5; MI WT females (n)= 7. Standard deviation is shown for wild type controls.

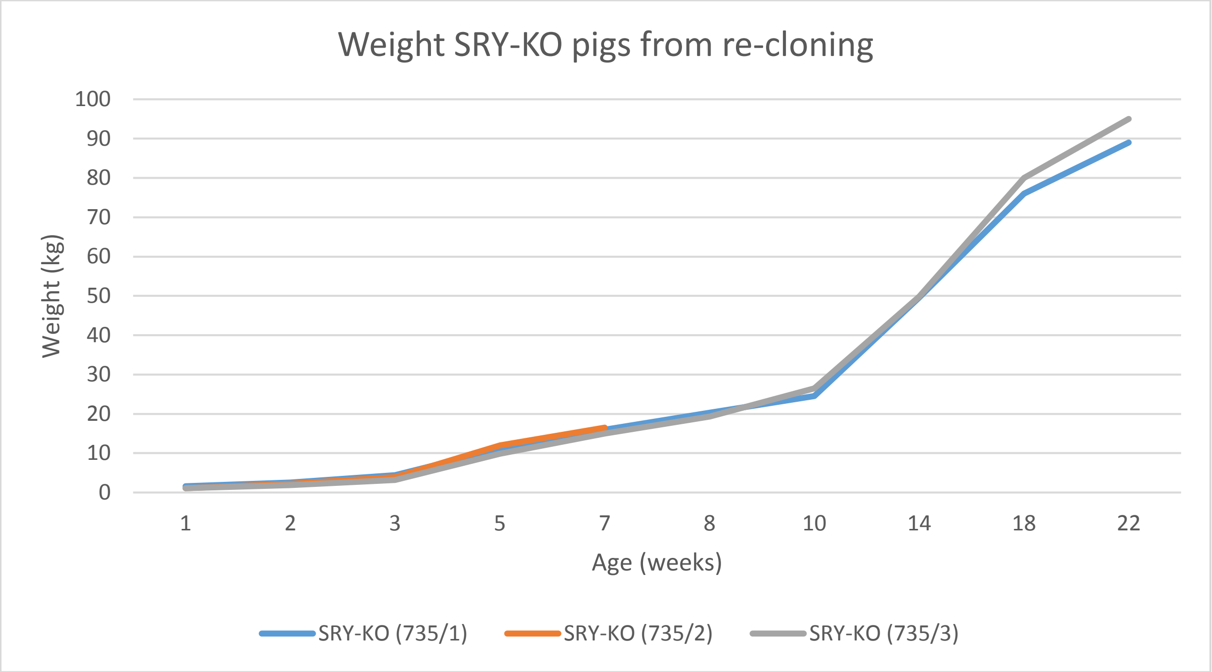

Figure S16 Schematic diagram showing the weight development of the SRY-KO pigs from re-cloning (735/1 to 3). The weights were determined at different time points during the first 22 weeks after birth. No statistical analysis was performed due to the low number of SRY-KO pigs (n=3, after week 7 n=2). SRY-KO pig 735/2 was euthanized with the age of two months to investigate the internal female genitalia.

A

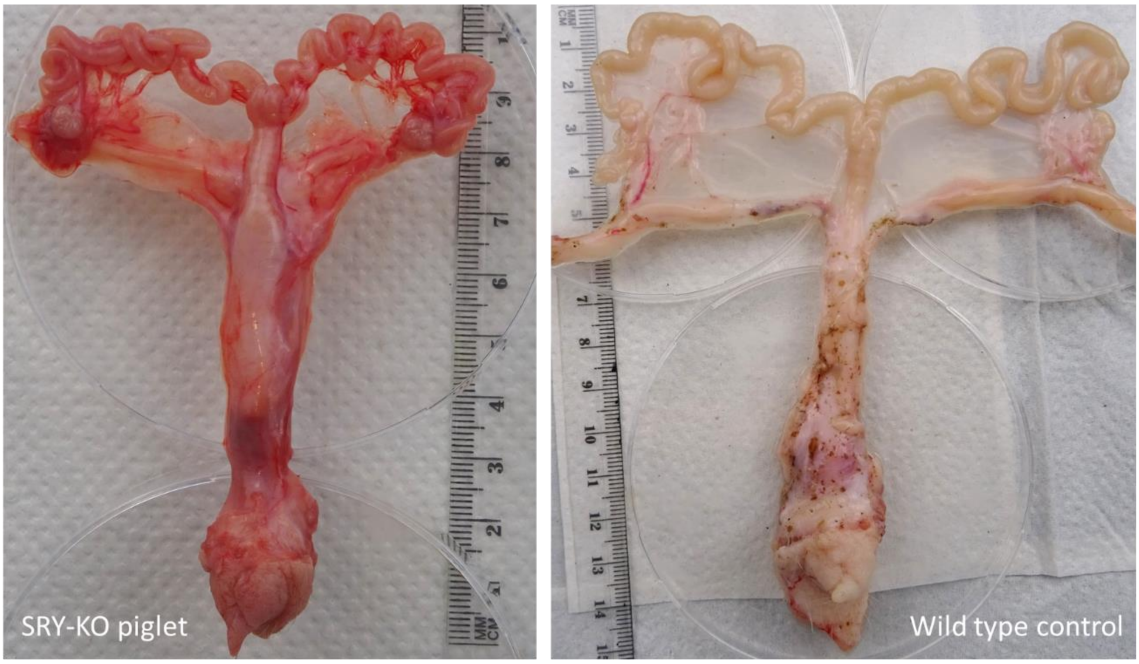

B

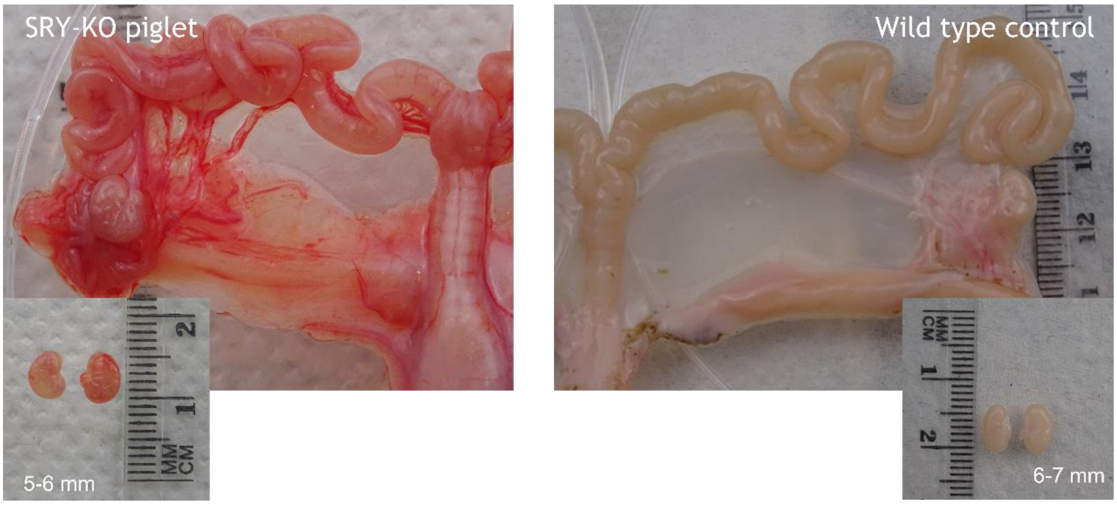

Figure S17 The uteri, oviducts and ovaries of the SRY-KO, XY piglet (715/2) and the WT, XX piglet (control from artificial insemination) at day 34. **a** No differences were detectable in size of oviduct and uteri. **b** Ovaries in SRY-KO, XY piglets are similar in size to ovaries of the WT, XX piglet.

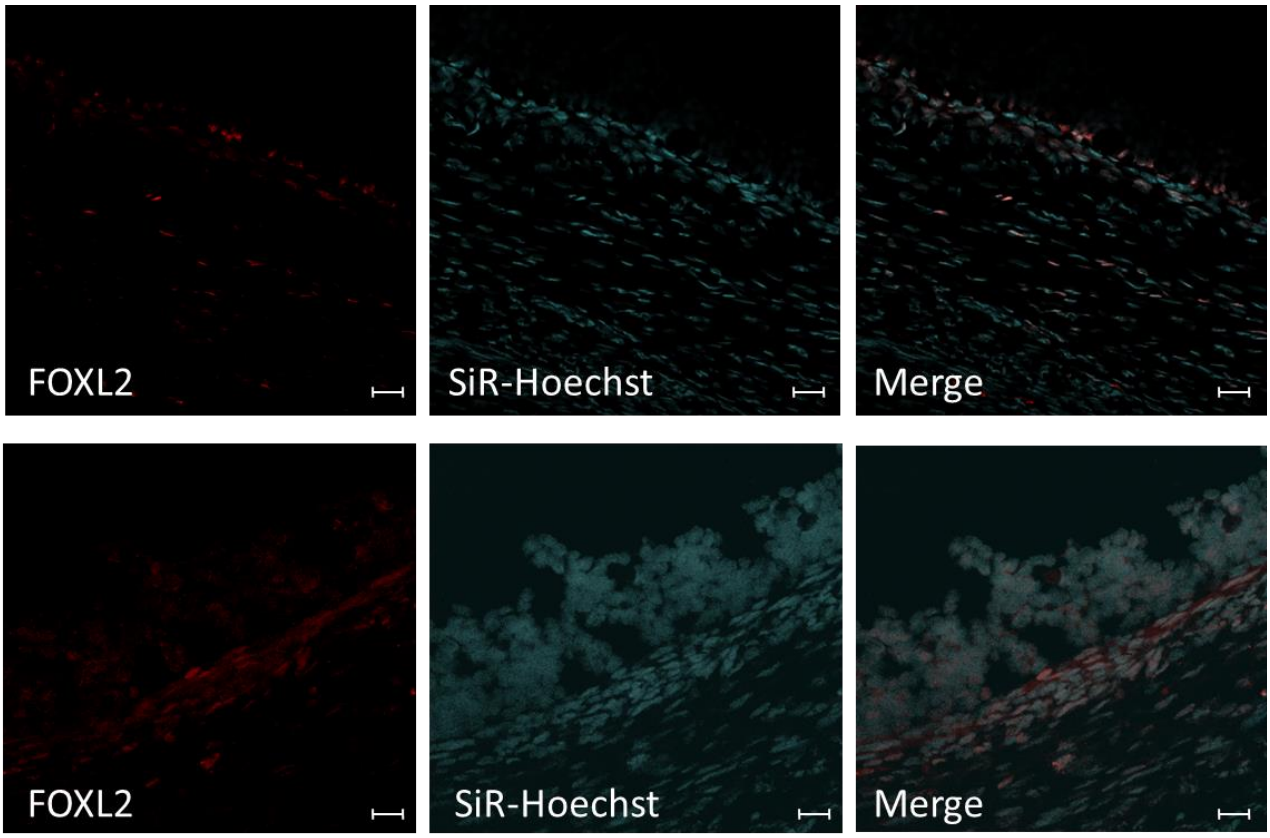

Figure S18 Immunohistological staining of FOXL2 positive cells (red) in ovaries of two 8-months old WT controls. Cell of FOXL2 positive cells were dispersed detected cortical regions of follicles of the porcine ovaries. SiR-Hoechst stained nuclei (blue). The merged images revealed positive FOXL2 staining in nuclei of the cells. The experiments were repeated independently three times with similar results. Scale bars, 20 µm.

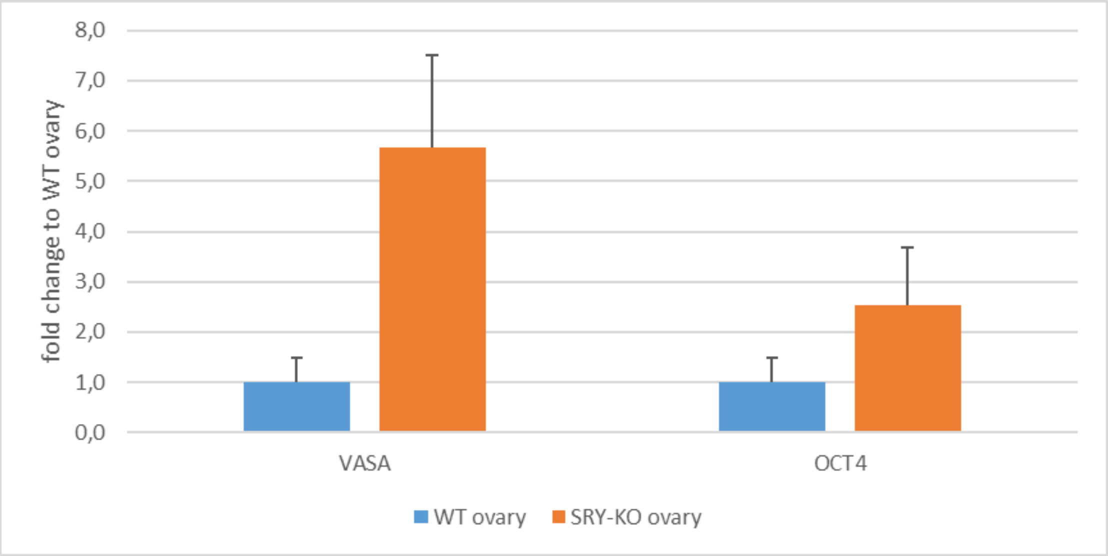

Figure S19 Real-time PCR analysis of ovaries isolated from SRY-KO pigs and female wild type controls for expression of VASA and OCT4 gene using pig-specific primers ("SI Appendix, Tab. S7"). Expression of VASA and OCT4 was 5.5-fold and 2.5-fold higher in SRY-KO pigs compared to the female WT controls. Fold change was calculated relative to expression of female WT controls. Technical replicates: wild type (WT) n=4, SRY-KO n=2

Table S1 Transfer of embryos generated by SCNT transfected with CRISPR/Cas plasmids SRY_1 and SRY_2 targeting the 5' flanking region of the HMG domain of the SRY gene. Two piglets were born with two different genetic modification (704/1 and 2). Both piglets displayed a male geno- and phenotype and no sex reversal.

**Recipient**	**Transferred embryos**	**Pregnancy**	**Offspring**	**Genetically male offspring**	**Genetic modification on the SRY gene**	**Sex reversal**
**7263 (704)**	82	+	2	2	2	-
**7266**	86	-	-	-	-	-

Table S2 For Y chromosome detection, six different Y chromosome specific genes (KDM6A, CUL4BY, DDX3Y, TXLINGY, UTY and UBA1Y) were utilized. Primer pairs for sequencing are listed.

Genloci	Primer	Sequence (5´- 3´)	Annealing Temp. (°C)	Length (bp)
KDM6A	KDM6A_1f	AAATCAAGGTATTACTTCACTATCCTG	60	442
	KDM6A_1r	ACCATGGAGATTTGACATCACCA		
CUL4BY	CUL4BY_1f	AGCCAGCCGGATAGAAAGTT	60	353
	CUL4BY_1r	ACAAAAGACACTCAGTTAAAACTTACC		
DDX3Y	DDX3Y_1f	ACTGGATTCTGTGTTCTTTGGA	60	291
	DDX3Y_2r	TTGGGGTGTTCTGTGCATGA		
TXLINGY	TXLINGY_1f	CCTCTAGCTTGTGGTTGGCA	60	249
	TXLINGY_1r	GTGGCCCTCTGAATCTTGCT		
UTY	UTY_1f	TCGTAAAGTGCTAAGTGGAGAAGA	60	345
	UTY_2r	TCAGGAACACACTGACGCTC		
UBA1Y	UBA1Y_1f	GCTGACACACTCACTGACCA	59	438
	UBA1Y_1r	AGCCATCAGAATCGTGTGGG		

Table S3 Top ten off-target sites for gRNA SRY_1 on the porcine genome. Primer pair for sequencing of the off-target events are listed. One off-target site could not be amplified.

Genloci	Primer	Sequence (5' – 3')	Annealing Temp. (°C)	Length (bp)
Chr. 5 - intergenic:	SRY_1.1_f	GAGGCTGAACTGGGAACCTT	60	2,509
XM_021091945.1	SRY_1.1_r	AGCACATGCTCTCTGCCAAA		
Chr. 18 - intergenic:	SRY_1.2_f	CATGCGCAGTCTGAACAAGG	58	3,134
XM_021079297.1	SRY_1.2_r	GGATGAAGGGTGCTAGACGG		
Chr. 4 - intron:	SRY_1.3_f	GAGCGCATCAACTGAGTGAC	65	1,227
XM_005663347.3	SRY_1.3_r	GGAAATGAGACAGGCCACCT		
Chr. 5 - intron:	SRY_1.4_f	CTCTGACTTGCACCCTGCTT	62	2,131
XM_021091957.1	SRY_1.4_r	ACTTCTCAATCCGCCCTATGC		
Chr. 3 - exon:	SRY_1.5_f	AACAACATGCGTCCAAACCG	62	1,776
XM_021086306.1	SRY_1.5_r	GCATCAGCACTCACCTGGAT		
Chr. 13 - exon:	SRY_1.6_f	AGTGACTGGGTTTGGGGTTG	62	2,002
XM_021069624.1	SRY_1.6_r	CGCCAGAGTCCCATACACTC		
Chr. 4 - intergenic:	SRY_1.7_f	-	-	-
XM_021090127.1	SRY_1.7_r	-		
Chr. 1 - intergenic:	SRY_1.8_f	CCTGACCACGTCGTATCTCG	61	3,035
NM_001114280.1	SRY_1.8_r	AGCTAAGGGTGGAGTTTGGC		
Chr. 8 - intergenic:	SRY_1.9_f	TAGCACCCCCAGAAACTCCT	62	2,602
XM_021101305.1	SRY_1.9_r	GGTGGATACTGTCAGCTGGG		
Chr. 16 - intergenic:	SRY_1.10_f	CCTAATTTGGCCTGCGCTTC	61	3,134
NM_001315615.1	SRY_1.10_r	ACCTCTGAGGGTGTGACCTT		

Table S4 Top ten off-target events for gRNA SRY_3 on the porcine genome. Primer pair for sequencing the off-target sites are listed. Three off-target sites could not be amplified.

Genloci	Primer	Sequence (5' – 3')	Annealing Temp. (°C)	Length (bp)
Chr. 14 - intergenic: XM_021073683.1	SRY_3.1_f	CTCCCCACAGCTGCTCTTTT	62	2,487
	SRY_3.1_r	GAATTGGGCACTTGCTGGAC		
Chr. 12 - intergenic: XM_003358165.4	SRY_3.2_f	-	-	-
	SRY_3.2_r	-		
Chr. 11 - intergenic: XM_021065302.1	SRY_3.3_f	AACAGGGAACCATCCACCAA	61	3,463
	SRY_3.3_r	CTCCAGGAGGCCATATGCTG		
Chr. 1 - intergenic: XM_021093786.1	SRY_3.4_f	-	-	-
	SRY_3.4_r	-		
Chr. 17 - intergenic: XM_003134254.5	SRY_3.5_f	AGCCTTATCCAATGAGGCCG	66	2,866
	SRY_3.5_r	CTAATGCCAGGGCAGTTTGC		
Chr. 13 - intron: XM_021070776.1	SRY_3.6_f	CAAAAGGCTACCAGGGGTGT	61	2,418
	SRY_3.6_r	GCCCAAGGTGACCTCAAACT		
Chr. 13 - intron: XM_021069725.1	SRY_3.7_f	-	-	-
	SRY_3.7_r	-		
Chr. 14 - intergenic: XM_013983698.2	SRY_3.8_f	CCCTGCCGTTAGATCCAGTC	66	4,663
	SRY_3.8_r	TCCAGAGGGCACCTGTGATA		
Chr. 4 - intron: XM_021090110.1	SRY_3.9_f	CTTCTCTGGTAACTGGCCCC	66	2,382
	SRY_3.9_r	TCCCGCAGATCCATTCCAAC		
Chr. 3 - intergenic: NM_001204768.1	SRY_3.10_f	CTTGTTCCTTCCTGGGTGGG	62	2,269
	SRY_3.10_r	CTCCAGATGGGGGACACTTG		

Table S5 Transfer of embryos generated by re-cloning of cells from piglet 715/2 transfected with CRISPR/Cas plasmids SRY_1 and SRY_3 into two recipients. Seven piglets (735/1 to 7) were born and all of them showed the sex reversal.

Recipient	Transferred embryos	Pregnancy	Offspring	Genetically male offspring	Genetic modification on the SRY gene	Sex reversal
**8117 (735)**	90	+	7	7	7	7
**8101**	93	-	-	-	-	-

Table S6 Growth weight (kg) of the SRY-KO pigs (715/2, 715/7 and 714/1) generated via intracytoplasmic microinjection compared to male (WT males) and female wild type controls (WT females) and female littermates from 1 to 36 weeks of age. The weight of each pig was determined once per week. Piglets 715/1, as wild type littermate control and SRY-KO piglet 715/2 were sacrificed on day 34 after birth to investigate the internal female genitalia. Growth weights of male and female WT controls were determined until weaning at the age of 8 weeks. The slaughter weight of two SRY-KO (715/7 and 714/1) and two MI WT (715/3 and 715/4) pigs was determined.

Pig	Number	Age (weeks)													
		1	2	3	4	5	6	7	8	9	10	11	12	13	36 (slaughter weight)
WT females weight (kg)	686-25	3	5.9	8	8	8	11.5	13.5	15.5	-	-	-	-	-	-
	686-26	3.2	5.8	8.3	8.2	9	11	14	15	-	-	-	-	-	-
	9000	3.8	6	9	9	10	11	13.9	17	21.5	25.5	29.6	34	-	-
	9001	2.9	5.6	8.5	8.4	8.5	8	-	-	-	-	-	-	-	-
	9002	1.8	3	5	5.1	5.5	9	9.5	11.5	14.5	18.5	23	27.4	-	-
WT males weight (kg)	686-19	2.9	4.6	8	8.1	8	10.5	12.1	15	-	-	-	-	-	-
	686-20	2.8	4.5	7	7	9	12	14	17	-	-	-	-	-	-
	686-21	3	4.9	7.2	6.9	8.5	12	13.9	16	-	-	-	-	-	-
	686-22	2.5	3.1	4.3	4	4.5	6.5	8	11	-	-	-	-	-	-
	9003	2.8	5	7.8	7	7	9	10	11.5	17.5	23	27.4	32	-	-
	686-24	3.1	6	9	9.1	9.5	11	14	16	-	-	-	-	-	-
MI WT females weight (kg)	715/1	-	5	7.1	10.8	-	-	-	-	-	-	-	-	-	-
	715/3	-	4.7	6.1	10.3	11	12.5	18.5	22.8	25.1	29.5	34	39.4	43.2	133.2
	715/4	-	4.9	6	9.9	10	10	15	19.1	20	24.5	29	33.5	38	133.8
	715/5	-	4	5.9	9	9	9	12	14.9	15.5	21.5	27	32	37.3	-
	715/6	-	5.1	7	10	10	10.5	13.5	17	17	20.5	23	28	33	-
	715/8	-	5	7.2	10.6	10.5	15.5	19.5	22.4	27	32.5	35.5	40.1	45	-
	715/9	-	6	9	12	12	16	20.5	25.4	27	33	38	42.6	47.2	-
	715/10	-	4,4	6	9.6	9.7	9	12.5	15	17	21	24	29	33.5	-
SRY-KO pigs weight (kg)	715/2	-	5.1	7	11	-	-	-	-	-	-	-	-	-	-
	715/7	-	4.4	6.9	9.7	10	13	17.5	21.9	24	30.5	34	39	43.5	137.9
	714/1	-	5.2	7	10.9	11	15	20.5	24.5	29	36.5	41	46.5	51	143.3

Table S7 Overview of the weight development (mean, median and standard derivation of growth rates) of the SRY-KO pigs (715/7 and 714/1) compared to male and female WT controls and female littermates (MI WT females) from 2 to 8 weeks of age. The pig weights were determined once per week and the mean, median and standard derivation of growth rates calculated per group. WT males (n)= 6; WT females (n)= 5; MI WT females (n)= 7;

	**WT males**			**WT females**			**WT MI females**			**SRY-KO (715/7)**	**SRY-KO (714/1)**
**Weight (kg) per week**	Mean	Median	Standard deviation	Mean	Median	Standard deviation	Mean	Median	Standard deviation		
2. Weeks	4.68	4.75	0.86	5.26	5.8	1.14	6.74	6.1	1.04	4.4	5.2
3. Weeks	7.22	7.5	1.45	7.76	8.3	1.42	10.2	10	0.87	6.9	7
4. Weeks	7.02	7	1.56	7.74	8.2	1.36	10.31	10	0.9	9.7	10.9
5. Weeks	7.75	8.25	1.65	8.2	8.5	1.5	11.79	10.5	2.74	10	11
6. Weeks	10.17	10.75	1.79	10.1	11	1.36	15.93	15	3.26	13	15
7. Weeks	12	13	2.3	12.76	13.7	1.87	19.51	19.1	3.82	17.5	20.5
8. Weeks	14.42	15.5	2.32	14.75	15.25	2.02	21.23	20	4.66	21.9	24.5

Table S8 Growth weight of the SRY-KO pigs (735/1 to 3) generated via re-cloning of cells from piglet 715/2 from week 1 to 22 of age. Piglet 735/2 was sacrificed at the age of 8 weeks to investigate the development of the internal female genitalia.

SRY-KO pig (weight in kg)	Age (weeks)									
	**1**	**2**	**3**	**5**	**7**	**8**	**10**	**14**	**18**	**22**
**735/1**	1.6	2.5	4.4	11.1	16	20.3	24.5	49.5	76	89
**735/2**	1.1	2.2	3.9	12	16.5	-	-	-	-	-
**735/3**	1.1	1.9	3.2	9.9	15	19.3	26.5	49.8	80	95

Table S9 List of the primers for real-time PCR. U: upper primer; l: lower primer; bp: base pairs

Genloci	Primer	Sequence (5' – 3')	Length (bp)
VASA: NM_001291682	pigVASA-u	CGGGTCGCTGTGGAAATACT	76
	pigVASA-l	AGTGGCTGTGCTAAATGGCT	
GAPDH: KJ786424.1	pigGAPDH-u	ACACTCACTCTTCTACCTTTG	89
	pigGAPDH-l	CAAATTCATTGTCGTACCAG	
EEF1A1: XM_005654402.3	pEEF1A1-u	CAAAAATGACCCACCTATGG	69
	pEEF1A1-l	GGCCAGGATGGTTCAGGATA	
OCT4: NM_001113060	pOCT4_ex4-5-u	AGTGCCCAAAGCCCACTCT	100
	pOCT4_ex4-5-l	TTCTGGCGACGGTTGCA	

Table S10 Digital PCR assay for GGTA on chromosome 1 and SRY and KDM6A on the Y chromosome.

Assay	Contains	Sequence 5' – 3'
GGTA1	Probe	CTGCGGACTCCTTCCGCCTCTC
	Primer Forward	CTCCTGAGTGATGTTTAGAACC
	Primer Reverse	ACATCCTGACGAGTTCACC
SRY	Probe	AGGATCGTGTCAAGCGACCCATGAAC
	Primer Forward	TGGACGTGAAACTGAGGAAG
	Primer Reverse	ACGAGACCACACAATGAAAG
KDM6A	Probe	TGTGTCTCCAACAAGCCTGATTGTTTC
	Primer Forward	GCCGAATGTGATTAGAAAGTCC
	Primer Reverse	TTTACCAAAGACAGCACACC
